# Early aggressive intervention for infantile atopic dermatitis to prevent development of food allergy: a multicenter, investigator-blinded, randomized, parallel group controlled trial (PACI Study)—protocol for a randomized controlled trial

**DOI:** 10.1186/s13601-018-0233-8

**Published:** 2018-11-23

**Authors:** Kiwako Yamamoto-Hanada, Tohru Kobayashi, Hywel C. Williams, Masashi Mikami, Mayako Saito-Abe, Kumiko Morita, Osamu Natsume, Miori Sato, Motoko Iwama, Yumiko Miyaji, Makiko Miyata, Shinichiro Inagaki, Fukuie Tatsuki, Narita Masami, Shoji F. Nakayama, Hiroshi Kido, Hirohisa Saito, Yukihiro Ohya

**Affiliations:** 1 Allergy Center, National Center for Child Health and Development, 2-10-1, Okura, Setagaya-ku, Tokyo, 157-8535 Japan; 2 Department of Management and Strategy, Clinical Research Center, National Center for Child Health and Development, Tokyo, Japan; 3 Centre of Evidence-Based Dermatology, University of Nottingham, Nottingham, UK; 4 Division of Biostatistics, Clinical Research Center, National Center for Child Health and Development, Tokyo, Japan; 5 Department of Pediatrics, School of Medicine, Keio University, Tokyo, Japan; 6 Department of Pediatrics, Hamamatsu University School of Medicine, Shizuoka, Japan; 7 Centre for Health and Environmental Risk Research, National Institute for Environmental Studies, Ibaraki, Japan; 8 Division of Enzyme Chemistry, Institute of Enzyme Research, Tokushima University, Tokushima, Japan; 9 Department of Allergy and Clinical Immunology, National Research Institute for Child Health and Development, Tokyo, Japan

**Keywords:** Atopic dermatitis, Prevention, Food allergy, Infants, Randomized controlled trial

## Abstract

**Background:**

Atopic dermatitis is the first clinical manifestation of the atopic march, with the highest incidence in the first year of life. Those affected often go on to develop other allergic diseases including food allergy, asthma, and allergic rhinitis. Recent evidence suggests that sensitization to foods may occur through a defective skin barrier which is common in atopic dermatitis in early life. We hypothesize that therapeutic aggressive intervention to treat new onset atopic dermatitis may prevent the development of later allergen sensitization, and associated food allergy, asthma, and allergic rhinitis.

**Methods:**

This study is a multi-center, pragmatic, two-parallel group, assessor-blind, superiority, individually randomized controlled trial. Atopic dermatitis infants (N = 650) 7–13 weeks old who develop an itchy rash within the previous 28 days are randomly assigned to the aggressive treatment or the conventional treatment in a 1:1 ratio. The primary outcome is oral food challenge-proven IgE-mediated hen’s egg allergy at the age of 28 weeks.

**Discussion:**

This is a novel pragmatic RCT study to examine the efficacy of early aggressive treatment for atopic dermatitis to prevent later food allergy. If our hypothesis is correct, we hope that such a strategy might impact on disease prevention in countries where food allergy is common, and that our results might reduce the frequency and associated costs of all food allergies as well as hens egg food allergy. Long-term follow and other similar studies will help to determine whether such a strategy will reduce the burden of other allergic diseases such as asthma and allergic rhinitis.

*Trial registration* UMIN-CTR: UMIN000028043

**Electronic supplementary material:**

The online version of this article (10.1186/s13601-018-0233-8) contains supplementary material, which is available to authorized users.

## Background

A systematic review of international trends in atopic dermatitis (AD) suggested that the prevalence of AD is increasing in Africa, eastern Asia, western Europe, and parts of northern Europe (e.g., the UK) [1]. According to a Japanese national survey, the prevalence of infants at the age of 6 months with AD or a suspected history of AD was about 25% [2].

The prevalence of food allergy (FA) has been increasing worldwide as well [3]. In Japan the most common food allergen was hen’s egg and the second most common was cow’s milk [4]. A general cohort study in Tokyo reported that the cumulative incidence of FA and hen’s egg allergy was 9% and 5.1%, respectively, among children aged 12 months [5].

Additionally, 50.8% of infants (95% CI 42.8, 58.9) with early AD onset (< 3 months old) who required doctor-prescribed topical corticosteroid treatment, who were more likely to be severe AD, developed challenge-proven FA [6]. Shoda et al. [5] demonstrated that in each age (by month) stratum, infants with onset of eczema within the first 1–2 months after birth had the highest risk of FA at 3 years of age (aOR 6.61; 95% CI 3.27, 13.34; *p *< 0.001) and the second highest was within 3–4 months after birth (aOR 4.69; 95% CI 2.17, 10.13). The results from previous studies [5, 6] suggest that infants who develop AD in early infancy have a higher risk for FA.

The allergic march describes the development of AD and concomitant sensitization to food and aeroallergens in early childhood, progressing to asthma and allergic rhinitis in later childhood or adult life [7]. A European cohort study noted that both early transient and early persistent AD increased the risk of food allergy at aged 6 (aOR 3.69; 95% CI 1.93, 7.035, and aOR 7.08; 95% CI 3.59, 13.975, respectively) [8]. In particular, early persistent AD increased the risk of asthma (aOR 2.87; 95% CI 1.31, 6.315), allergic rhinitis (aOR 4.04; 95% CI 1.82, 8.955) and sensitization to nhalant allergens (aOR 3.36; 95% CI 1.78, 6.355) at aged 6. Martin et al. [6] reported that 20% of 1-year-old infants who had a history of eczema received a diagnosis of FA.

Lack et al. [9] suggested the dual-allergen-exposure hypothesis which states that tolerance is induced from oral exposure to food antigen, enhancing immune response to suppress allergy and that infants with eczema are exposed to food antigen via skin and induce immune cells, enhancing allergy and producing IgE antibodies (sensitization). This hypothesis implies that it is important not only to induce oral immune tolerance, but to prevent allergic sensitization through the skin to reduce allergies. Inflammation of the skin and allergic sensitization is suggested as a mechanism of AD. Lack et al. [10] demonstrated that peanut allergy was positively associated with the use of skin care products containing peanut oil in England. The results from the study suggested that peanut allergens absorbed through the skin may cause allergic sensitization.

Topical corticosteroids (TCSs) and tacrolimus ointments are the mainstream of pharmacotherapy to suppress skin inflammation associated with AD. The approach for using TCSs in AD is either reactive or proactive management [11, 12]. Reactive management is when TCSs are administered only when the rash worsens. Proactive management is applying anti-inflammatory TCS therapy intermittently even after the skin clears to suppress subclinical inflammation of the skin in AD patients. Fukuie et al. [13] performed a 2-year retrospective cohort study of patients with moderate to severe AD to investigate whether proactive management changes serum IgE level compared to reactive management. Serum total IgE titer was significantly decreased in the proactive treatment group compared with the reactive treatment group (2442 IU/mL vs. 2081 IU/mL; *p *< 0.01). In addition, the serum egg white-specific IgE level decreased significantly during follow-up (60.0 IU/mL vs. 36.6 IU/mL; *p *= 0.004). In a case control study, infants with early proactive management (beginning at ≤ 4 months old) had a lower prevalence of egg allergy compared to infants with later proactive management (beginning at ≥ 5 months) at the age of 18 months (9.1% vs. 24.2%) [14]. Previously published results suggest that proactive management might be effective for reducing eczema flare-ups, and for preventing development of allergic sensitization. Thus, early proactive management for infantile AD may reduce the risk of FA.

Natsume et al. [15] conducted a double-blind, placebo-controlled RCT (PETIT Study) for infants 4–5 months of age with eczema who were enrolled and randomly assigned to the early introduction of egg or placebo to examine the proportion of participants with hen’s egg allergy confirmed by open oral food challenges at 12 months of age. In the study, 121 participants revealed that the prevalence of egg allergy was 37.7% in the placebo group (n = 61) and 8.3% in the egg group (n = 60), and the risk ratio was 0.221 (95% CI 0.090, 0.543; *p *= 0.00013); this demonstrated that the prevalence in the egg group was significantly lower than that in the placebo group. Especially, there were “zero” infants with hen’s egg allergy at 12 months of age among the infants who were not sensitize to hen’s egg at entry of the study. Immune tolerance is not considered to be induced merely by orally taking allergenic foods from the early stage of infancy.

Looking at allergic march, AD is the first clinical manifestation with the highest incidence in the first year of life and those affected develop other allergic diseases such as FA, asthma, and allergic rhinitis later in childhood in most patients. We think that it is important not only to introduce oral immune tolerance by early hen’s egg consumption, but to prevent sensitization via skin to prevent the development of FA. We hypothesize that to prevent future allergic march, an appropriate intervention for AD, which emerges at the first stage of allergic march, is important to be considered. We expect that early aggressive intervention with proactive method for AD will likely prevent development of later allergen sensitization, FA, asthma, and allergic rhinitis. The main purpose is to test by a randomized controlled trial the superiority of aggressive intervention over conventional treatment of infantile AD to prevent FA.

## Methods/design

**Fig. 1:**
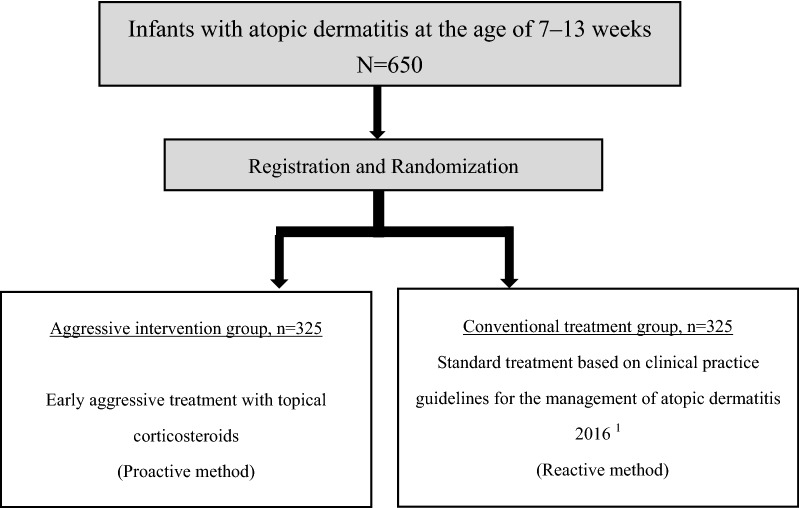
Trial scheme

This study is a multi-center, pragmatic, two-parallel group, assessor-blind, superiority, individually randomized controlled trial (Fig. 1). The study intervention is continued until participants become 6 months old. Primary endpoint is assessed at the time when participants become 6 months old.

### Setting

The study is recruiting infants with atopic dermatitis across Japan. Investigational sites include tertiary care centers that are found in the acknowledgement.

### Participants

Infants with AD (N = 650) who meet all of the following inclusion criteria and none of the exclusion criteria will be enrolled in PACI Study.

### Inclusion criteria

Infants must meet the following criteria to be enrolled in the study:

1. Infants (7–13 weeks old) who develop an itchy rash within the previous 28 days and receive a diagnosis of AD based on the under 4 years old version of the U.K. Working Party’s diagnostic criteria [16–18].

### Exclusion criteria

Infants who meet any of the following will be excluded from the study:

1. Infants born before 37 weeks of gestation
2. Twin or multiple
3. History of emollient (heparinoid cream: Hirudoid^®^ Soft ointment) and TCSs (alclometasone dipropionate: Almeta^®^; betamethasone valerate: Rinderon^®^-V; mometasone furoate: Fulumeta^®^) side effects
4. History of taking oral or intravenous steroids within the previous 28 days
5. History of taking immunosuppressive agents (cyclosporine, tacrolimus, and so forth) or biologics, except vaccinations or intravenous immunoglobulins, within the previous 28 days
6. IgE-mediated hen’s egg allergy
7. Infants whose immediate family plans to move and who may not be able to visit the study site at 28 weeks of age
8. Parents unable to understand Japanese
9. Unwillingness to adhere to the study requirements and procedures
10. Infants with severe disease and other skin diseases that affect dermatological evaluation and study physicians judge that they are not appropriate for study participation

### Interventions

Participants apply the emollients and TCSs described below as study drugs.

Emollients: Heparinoid cream (Hirudoid^®^ Soft ointment)

TCSs: Alclometasone dipropionate (Almeta^®^), betamethasone valerate (Rinderon^®^-V), and mometasone furoate (Fulumeta^®^)

Participants in aggressive intervention group obtain Early aggressive treatments with topical corticosteroids (proactive method). Hanifin et al. [19] performed an RCT for participants who were at least 3 months old and who had moderate or severe AD, to examine the efficacy of proactive therapy. Early aggressive treatments are modified from the Hanifin et al. study intervention, and details are described in Additional file 1. On the other hand, participants in conventional treatment group obtain standard treatment based on the Guidelines for the Management of Atopic Dermatitis (2016) [20] (Step-up reactive method). Standard treatment is based on the Guidelines for the Management of Atopic Dermatitis (2016) [20], and the details are described in Additional file 1.

### Aggressive intervention group

#### Basic whole-body treatment

Participants will be followed as described below and administered basic whole-body treatment, except for scalp.

Emollients

**Table:** 

	Whole body except scalp
Registration day (Day 0) of the study to 28 weeks of age	Hirudoid^®^ Soft ointment every day twice a day

Topical corticosteroids

**Table:** 

	Face	Body except scalp and face
Registration day (Day 0) to Day 14 of the study	Almeta^®^ ointment every day twice a day	Rinderon^®^-V ointment every day twice a day
Day 15 of the study to 28 weeks of age	Almeta^®^ ointment two days per week twice a day	Rinderon^®^-V ointment 2 days per week twice a day

#### Additional skin rash treatment

Participants are to apply TCSs as additional treatment as described below:

**Table:** 

Face	Body except scalp and face	Scalp
Day 15 of the study to 28 weeks of age	Day 15 of the study to 28 weeks of age	Day 15 of the study to 28 weeks of age
Almeta^®^ ointment every day until rash remission twice daily	Rinderon^®^-V ointment every day until rash remission twice a day	Rinderon^®^-V lotion until rash remission twice a day

### Conventional treatment group

#### Basic whole-body treatment

Participants will be followed as described below and administered basic whole-body treatment, except for scalp.

**Table:** 

	Whole body except scalp
Registration day (Day 0) of the study to 28 weeks of age	Hirudoid^®^ Soft ointment every day twice a day

#### Additional skin rash treatment

Participants are to apply TCSs as additional treatment as described below:

**Table:** 

Area	Face		Body except scalp and face			Scalp
Severity of skin rash	Less mild	Mild, moderate, and severe	Less mild	Mild and moderate	Severe	Any severity
Registration day (Day 0) of the study to 28 weeks of age	Without additional treatment	Almeta^®^ ointment every day until rash remission once a day	Without additional treatment	Almeta^®^ ointment every day until rash remission once a day	Rinderon^®^-V ointment every day until rash remission once a day	Rinderon^®^-V lotion until rash remission once a day

Proxies (parents or legal guardians) of the participants will be taught how to use the TCSs and the emollient for their infants in each group by a study physician with video lectures and skin care leaflets. Nutritional education for participants will also be given on the day of study registration.

It is recommended to introduce solid food to infants at 4–5 months of age. Breastfeeding is encouraged to continue until at least 6 months of age. Mothers who are breastfeeding are not restricted from ingesting hen’s egg. Participants are not permitted to eat hen’s egg until the oral food challenge test at the age of 28 weeks is completed.

### Primary outcome

Primary outcome is a presence of oral food challenge-proven IgE-mediated hen’s egg allergy at the age of 28 weeks because hen’s egg is the most common causal FA in Japan.

The timing of oral food challenge tests for hen’s egg is set at the age of 28 weeks because we decided to make a recommendation for participants in our study to ingest hen’s egg starting at 6 months of age based on the past study results reported by PETIT Study [15].

### Secondary outcomes

Efficacy endpoints include the following:

1. Food challenge test scores [21] at the age of 28 weeks
2. Total IgE antibody titre in serum at the age of 28 weeks
3. Egg white, ovomucoid, milk, soy, wheat, and peanut-specific IgE antibody titers in serum at the age of 28 weeks
4. Egg white, ovomucoid, milk, soy, wheat, and peanut-specific IgG4 antibody titers in serum at the age of 28 weeks
5. Eczema Area and Severity Index (EASI) scores [22] at 2, 4, and 8 weeks after study entry and at the age of 28 weeks
6. Patient Oriented Eczema Measure (POEM) scores [23] weekly through the study
7. Percentage of disease-free days throughout the study
8. Dose of rescue medication used throughout the duration of the study
9. Infants’ Dermatitis Quality of Life Questionnaire (IDQoL) [24, 25] at 2, 4, and 8 weeks after study entry and at 28 weeks of age
10. Family Impact of Childhood Eczema Questionnaire (DFI) [26] at 2, 4, and 8 weeks after study entry and at 28 weeks of age
11. Cumulative incidence of IgE-mediated FA assessed by a doctor’s interview during the study
12. Cumulative incidence of wheezing assessed by a doctor’s interview during the study (parental reports evaluated by doctor)

Safety endpoints include the following:

1. Serious adverse events
2. Adverse drug reactions
3. Salivary cortisol concentrations at 2, 4, and 8 weeks after study entry and at the age of 28 weeks
4. Body weight and body height at the age of 28 weeks

Adherence endpoints include the following:

1. TCS adherence index in accordance with the protocol
2. Dose of TCS used during the study

### Study procedures

This study uses VIEDOC™ selected as the Electronic Data Capture system (EDC) by Pharma Consulting Group Japan K.K. to register and allocate participants and perform data collection. Study physicians confirm eligibility criteria. Based on the result of the confirmation of eligibility criteria, study physicians obtain informed consent for study participation using a fixed document request from the proxy (parent or legal guardian) of the participants who meet all of the inclusion criteria and none of the exclusion criteria. Stratified block assignment with the number of weeks after birth (7–10 weeks, 11–13 weeks) as the factor in the order of registration is performed and participants are randomly assigned to the “aggressive intervention group” or “conventional treatment group” in a ratio of 1:1 using EDC system (VIEDOC™). Randomization sequence is generated by VIEDOC™. Earlier the onset of eczema is, higher the incidence of food allergy becomes in later life [5, 6], so the number of weeks after birth was assigned as a stratification factor. Study assessment schedule is shown in Table 1. Food challenge test (hen’s egg) is open method to participants and their caregivers and assessed by blinded physician, who do not know participants’ group assigned nor give treatment to them, at the age of 28 weeks. Pasteurized low egg powder 2.6 g (whole egg 10.4 g, egg protein 1.2 g) is given. Participants eat 0.1–0.5–2 g of the pasteurized egg powder every 40 min. The challenge tests are evaluated based on the PRACTALL criteria [21],

**Table 1:**
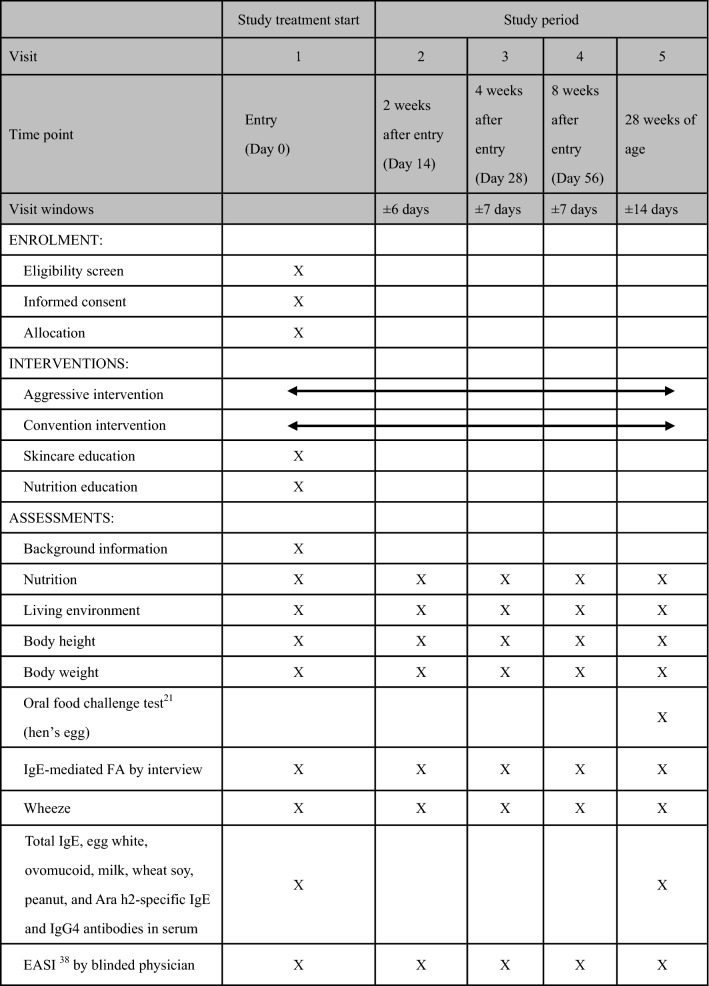

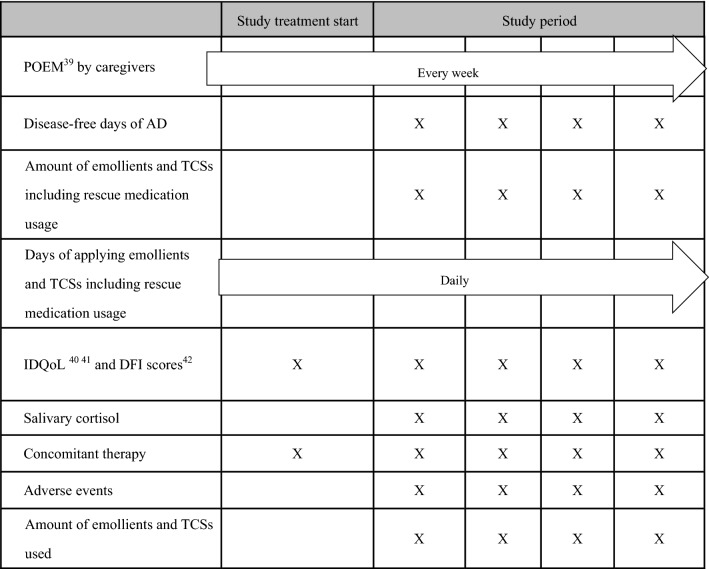
Assessment schedule

Participants, parents, and investigators who treat participants with AD are not blinded to the study intervention. The physicians who perform OFC and examine EASI are blinded to the group allocations. Those who are blinded to the study intervention are required to meet the following criteria.

1. Blinded physicians are not the physicians who treat participants in the PACI Study.
2. Blinded physicians do not look at documents and all other information about the study intervention.

Study physicians obtain the most recent information about the study medicines (Hirudoid^®^ Soft ointment, Almeta^®^, Rinderon^®^-V, and Fulumeta^®^) from their package inserts. We evaluate adverse drug reaction. Participants can discontinue the study if participants have adverse events (see 5.9.2 STUDY DISCONTINUATION on Additional file 1).

### Sample size calculation

Palmer et al. [27] performed a double-blind, randomized controlled trial for infants with AD to test whether or not one-sixth of a raw whole egg taken every day can prevent development of IgE-mediated egg allergy. The infants were allocated to receive 1 teaspoon of pasteurized raw whole egg powder (n = 49) or rice powder (n = 37) daily from 4 to 8 months of age. A high proportion (31% [15/49]) of infants randomized to receive egg had an allergic reaction to the egg powder and 20% (10/49) had a reaction at the first ingestion of egg powder (one-sixth of a raw whole egg) at 4 months of age. When the control group participants received half of a raw egg provocation test at 12 months, 51% (18/35) were diagnosed with IgE-mediated egg allergy. These data suggested that prevalence of egg allergy in infancy is likely to depend on the provocation dose of egg and the preparation of egg such as heated or raw, and also on age and race of the participant. Therefore, we estimate that at least 30% of the participants in the conventional treatment group in this study will be diagnosed with IgE-mediated hen’s egg allergy using an oral food challenge test at the age of 28 weeks. However, the prevalence of IgE-mediated hen’s egg allergy among infants treated with early aggressive intervention for AD has never been reported. In Japan, it is common that infants at the age of 28 weeks have not eaten hen’s eggs, and the prevalence of hen’s egg allergy at the age of 28 weeks in Japan is unclear. We consider a clinically effective prevalence of infants with early aggressive intervention for AD to be 20%. For sample size calculation, the proportion of egg allergy in early aggressive intervention and conventional treatment groups are estimated to be 20% and 30%, respectively. With a one-sided significance level of 0.025, 581 participants are needed to provide 80% statistical power. One interim analysis examining efficacy is planned when almost half the participants are finished their trial assessment. Considering the interim analysis, 614 participants are needed. We expect a drop of about 5% and set the target number of study participants to 650.

### Statistical analysis

Statistical analysis will be performed following the intention to treat (ITT) principle, where participants will be analyzed as they were randomized, not as they were treated. The primary analysis is performed using full analysis set (FAS), and secondarily using per protocol set (PPS). The participants who have missing data for the presence of IgE-mediated hen’s egg allergy are defined as having IgE-mediated hen’s egg allergy and included in the FAS analysis. The participants who have missing data for the presence of IgE-mediated hen’s egg allergy are excluded from the PPS analysis. To verify the study hypothesis, “the aggressive intervention group has lower prevalence of IgE-mediated hen’s egg allergy compared to the conventional treatment group”, the main comparison is the null hypothesis that the percentage of IgE-mediated hen’s egg allergy is higher in the aggressive treatment group than in the conventional group” and the alternative hypothesis is that the percentage of IgE-mediated hen’s egg allergy is lower in the aggressive treatment group than in the conventional group. The *p* value is calculated by the difference in percentage with a one-sided test. The significance level is 0.025. The difference and ratio of these proportions and their 95% confidence intervals for each intervention group will also be calculated. All secondary endpoints will be analyzed using FAS and secondarily using PPS. The details of statistical methods are described in the statistical analysis plan.

Interim analysis is conducted with the aim of preventing disadvantages to participants due to excessive efficacy procedures unintended in study treatment. The interim analysis is performed when almost half of the participants finish their assessments for the primary endpoint. The interim analysis is done for primary endpoint and safety endpoints. The study registration for study participants does not stop while conducting the interim analysis. The analysis of primary endpoint is performed along with the primary endpoints analysis. The stopping boundary for the analysis of primary endpoint will be calculated based on the O’Brien–Fleming type with a Lan–DeMets α and βspending function at the actual information time. For example, the significance levels of effective discontinuation and invalid discontinuation are 0.0015 and 0.2883, respectively, with the interim analysis for 307 participants who are half of the planned study participants. The interim analysis is conducted by a trial statistician of the interim analysis under a closed situation. The statistician prepares a report of interim analysis results and submits it to the independent data monitoring committee. The results of the interim analysis will be evaluated by the members of the independent data monitoring committee to investigate whether or not the study continues, and the investigation result report is submitted to the principle investigator.

### Monitoring and ethics

This study follows the Declaration of Helsinki Ethical Principles for Medical Research Involving Human Subjects and the Ethical Guidelines for Medical and Health Research Involving Human Subjects (2014 December 22, the Japanese Ministry of Education, Ministry of Health, Labour and Welfare Notification No. 3). PACI Study has been approved by IRB of National Center for Child Health and Development (No. 1347) and each investigational site. Details are described in Additional file 1.

## Discussion

### Potential benefits of study

To our knowledge, this would be the first pragmatic RCT study to examine the efficacy of early aggressive intervention for AD to prevent later FA. If we establish a novel new strategy for prevention of FA development by early aggressive intervention for AD, it is expected that quality of life in patients will improve, the prevalence of FA will decrease, and the medical care cost will be reduced. In addition, we are doing follow-up cohort study, PACI-On Study, after the PACI Study. We will observe the participants until they reach to 6 years old. From the results of the PACI-On Study, we will reveal that natural course of allergic diseases could be changed by this early aggressive intervention.

### Potential study harms

Although we use TCSs approved officially in daily basis, we should consider the safety of TCSs. TCSs are topical anti-inflammatory agents and they are safer than systemic steroids administered via an oral or intravenous route. Absorption of TCS in the skin is considered to depend on many factors such as molecular weight of TCS, cream or ointment bases, application amount, potency of TCS, application period, and age. Cutaneous side effects include telangiectasias, skin atrophy, skin striae, focal hypertrichosis, acne-like eruptions, rosacea-like eruptions, folliculitis, and so forth [28, 29]. In an observational study in the US, children with moderate or severe AD did not show adrenal suppression although they had used TCSs for more than several years since infancy [30]. Fukuie et al. [19] performed rapid ACTH stimulation tests for eight children in the proactive group and four children in the reactive group at 3 months after starting the study intervention and found that no children showed adrenal suppression. However, Hanifin et al. [31] demonstrated that 2/44 children in the proactive group had adrenal suppression in the RCT. Children are more susceptible to steroids than adults and, therefore, their treatment should be carefully considered.

### Other similar studies

Looking into prevention of AD by moisturizer, two randomized controlled trials (RCTs) were performed to investigate whether protecting the skin barrier with a moisturizer applied at the beginning of the neonatal period would prevent development of infantile AD. These studies demonstrated that application of a moisturizer during early life reduced the incidence of AD/eczema in infants [32, 33]. However, the studies could not confirm the efficacy of food allergy prevention. However, BEEP Study is ongoing RCT in the UK to investigate whether daily application of emollients for the first year of life can prevent not only AD, but FA and asthma developing in high-risk infants [34]. BEEP Study will validate the efficacy of emollients for prevention of FA in the future. PreventADALL study is also ongoing, a 2 × 2 factorial, RCT to examine the efficacy of primary prevention of allergic diseases such as atopic dermatitis and food allergy by regular intake of six-food-items from 3 months of age and emollient in general populations of Norway and Sweden [35]. Shimojo et al. conducted a 2 × 2 factorial RCT to investigate whether emollient application and synbiotics could prevent atopic dermatitis and food allergy in newborn babies in Japan (UMIN-CTR: UMIN000010838). Several combinations of interventions might be effective to prevent allergy development.

### Importance of disease prevention studies

A systematic review demonstrated that the pooled lifetime and point prevalence of self-reported FA was 17.3% (95% CI 17.0–17.6) in Europe [36]. From a large-scale birth cohort study (JECS Study), the prevalences of asthma, allergic rhinitis (hay fever), atopic dermatitis, and food allergy were 10.9, 36.0, 15.7 and 4.8%, respectively, among 99,013 mothers in Japan [37]. In addition, 73.9% mothers had positive IgE sensitization. In 2015, the Japanese Ministry of Health, Labour and Welfare reported that 3 of the most common diseases in outpatients younger than 15 years were allergic rhinitis, asthma, and atopic dermatitis [38]. In addition, a government report from 2013 documented a marked increase in food allergy in school children in Japan [39]. Allergic diseases are serious health concerns and economic burdens globaly [40]. Patients with FA often have a lower quality of life as a result of restrictions on eating causal food, which is required because of their high risk of developing anaphylaxis [41]. Therapeutic strategy to prevent allergy such as food allergy needs to be developed [42]. As we mentioned before, to prevent future allergic march, an appropriate intervention for AD, which emerges at the first stage of allergic march, is important to be considered. We expect that early aggressive intervention for AD will likely prevent development of later allergen sensitization, FA, asthma, and allergic rhinitis. If we establish a novel new strategy for prevention of FA development by early aggressive intervention for AD, it is expected that quality of life in patients will improve, the prevalence of FA will decrease, and the medical care cost will be reduced.

## Additional file

**Additional file 1.** PACI Study Protocol version 1.2.

## Abbreviations

AD: atopic dermatitis
aOR: adjusted odds ratio
CI: confidence interval
CTR: clinical trial registry
EASI: Eczema Area and Severity Index
EDC: electronic data capture
FA: food allergy
DFI: Family Impact of Childhood Eczema Questionnaire
IDQoL: Infants’ Dermatitis Quality of Life Questionnaire
IgE: immunoglobulin E
IgG: immunoglobulin G
IRB: Institutional Review Board
ITT: intention to treat
NCCHD: National Center for Child Health and Development
OR: odds ratio
OFC: oral food challenge
PACI Study: Prevention of Allergy via Cutaneous Intervention Study
PETIT Study: Prevention of Egg Allergy with Tiny Amount Intake Trial
POEM: patient-oriented eczema measure
PP: per protocol
RCT: randomized controlled trial
TCSs: topical corticosteroids
UK: United Kingdom
US: United States of America
UMIN-CTR: University Hospital Medical Information Network
