# Supplementary material for: Early aggressive intervention for infantile atopic dermatitis to prevent development of food allergy: a multicenter, investigator-blinded, randomized, parallel group controlled trial (PACI Study)—protocol for a randomized controlled trial
Source: Clin Transl Allergy. 2018 Nov 23;8:47. doi: 10.1186/s13601-018-0233-8 (PMC6251129; doi:10.1186/s13601-018-0233-8)
Supplement: Supplementary file 1 — Additional file 1. PACI Study Protocol version 1.2. [file 13601_2018_233_MOESM1_ESM.docx]

The Japan Agency for Medical Research and Development (AMED)

Practical Research Project for Allergic Diseases and Immunology (Research on Allergic Diseases and Immunology)

Research group “Early Intervention Study for Infantile Atopic Dermatitis to Prevent Allergic March Development”

**Early Aggressive Intervention for Infantile Atopic Dermatitis to Prevent Development of Food Allergy – a Multicenter, Investigator-Blinded,**

**Randomized, Parallel Group Controlled Trial**

**Short Title: Prevention of Allergy via Cutaneous Intervention (PACI) study**

**Trial protocol**

**Ver1.2**

lRB Approval: IRB of National Center for Child Health and Development (No. 1347)

Trial registration: UMIN-CTR (000028043)

Funding Source: The Japan Agency for Medical Research and Development (AMED)

ESTABLISHMENT AND AMENDMENT

| Version | Day of Amendment | Implemented by | Details |
| --- | --- | --- | --- |
| 1.00 | 2017/2/27 | Kiwako Yamamoto-Hanada | Approval by IRB of NCCHD |
| 1.10 | 2017/3/24 | Kiwako Yamamoto-Hanada | 0.8 contact information revised, 5.2 registration revised  6.1 study schedule revised, 10.0 data management revised, 13.6 commissioned business and commissioned project and provision of commissioned office deleted |
| 1.10 | 2017/6/12 | Kiwako Yamamoto-Hanada | Group name revised from early aggressive intervention to aggressive intervention (reporting to IRB and approved by IRB via documentation) |
| 1.11 | 2017/7/18 | Kiwako Yamamoto-Hanada | Members of monitoring group revised, member*s* of the data center and central monitors revised, and investigational site added, supplement 12 person in charge of the data center changed |
| 1.2 | 2018/2/8 | Kiwako Yamamoto-Hanada | Investigational sites added |

TABLE OF CONTENTS

[0 PROTOCOL SYNOPSIS 6](#_Toc513157082)

[0.1 STUDY SCHEME 6](#_Toc513157083)

[0.2 PURPOSE 6](#_Toc513157084)

[0.3 STUDY DESIGN 6](#_Toc513157085)

[0.4 PARTICIPANTS 6](#_Toc513157086)

[0.5 PRIMARY ENDPOINT 6](#_Toc513157087)

[0.6 STUDY PROCEDURE 6](#_Toc513157088)

[0.7 ANTICIPATED NUMBER OF PARTICIPANTS AND STUDY DURATION 8](#_Toc513157089)

[0.8 CONTACT INFORMATION 8](#_Toc513157090)

[1 BACKGROUND 10](#_Toc513157091)

[1.1 ATOPIC DERMATITIS 10](#_Toc513157092)

[1.2 FOOD ALLERGY 14](#_Toc513157093)

[1.3 EXPECTATIONS FOR EARLY AGGRESSIVE INTERVENTION FOR ATOPIC DERMATITIS 18](#_Toc513157094)

[1.4 NECESSITY AND SIGNIFICANCE OF THE STUDY 18](#_Toc513157095)

[2 PURPOSE 18](#_Toc513157096)

[2.1 STUDY HYPHOTHESIS 18](#_Toc513157097)

[3 DEFINITIONS 19](#_Toc513157098)

[4 ELIGIBILITY 23](#_Toc513157099)

[4.1 INCLUSION CRITERIA 23](#_Toc513157100)

[4.2 EXCLUSION CRITERIA 24](#_Toc513157101)

[5 STUDY PROCEDURES 24](#_Toc513157102)

[5.1 STUDY DESIGN 24](#_Toc513157103)

[5.2 REGISTRATION 25](#_Toc513157104)

[5.3 RANDOMIZATION AND STRATIFICATION 26](#_Toc513157105)

[5.4 BLINDING 26](#_Toc513157106)

[5.5 PATIENT BACKGROUND SURVEY AND START OF STUDY TREATMENT 27](#_Toc513157107)

[5.6 STUDY INTERVENTION 27](#_Toc513157108)

[5.7 PERMITTED CONCOMITANT TREATMENT 29](#_Toc513157109)

[5.8 NON-PERMITTED CONCOMITANT TREATMENT 30](#_Toc513157110)

[5.9 STUDY COMPLETION AND DISCONTINUATION 30](#_Toc513157111)

[6 STUDY ASSESSMENTS (Observation, test, and evaluation) 30](#_Toc513157112)

[6.1 ASSESSMENT SCHEDULE 30](#_Toc513157113)

[6.2 ASSESSMENT DETAILS 32](#_Toc513157114)

[7 ADVERSE EVENTS 33](#_Toc513157115)

[7.1 COLLECTION OF ADVERSE EVENTS 33](#_Toc513157116)

[7.2 ASSESSMENT OF ADVERSE EVENTS 33](#_Toc513157117)

[7.3 REPORTING OF ADVERSE EVENTS 35](#_Toc513157118)

[8 ENDPOINTS 35](#_Toc513157119)

[8.1 PRIMARY ENDPOINT 35](#_Toc513157120)

[8.2 SECONDARY ENDPOINTS 36](#_Toc513157121)

[9 STATISTICS 37](#_Toc513157122)

[9.1 SAMPLE SIZE 37](#_Toc513157123)

[9.2 ANALYSIS SET 37](#_Toc513157124)

[9.3 STATISTICAL ANALYSIS 37](#_Toc513157125)

[10 DATA MANAGEMENT 38](#_Toc513157126)

[11 ETHICAL CONSIDERATIONS 38](#_Toc513157127)

[11.1 ETHICAL REVIEW 39](#_Toc513157128)

[11.2 INFORMED CONSENT 39](#_Toc513157129)

[11.3 WITHDRAWAL OF CONSENT 39](#_Toc513157130)

[11.4 PRIVACY AND CONFIDENTIALTY 40](#_Toc513157131)

[11.5 EXPECTED BENEFITS AND DISADVANTAGES 40](#_Toc513157132)

[12 PROTOCOL VIOLATIONS AND DEVIATIONS 41](#_Toc513157133)

[12.1 VIOLATIONS 41](#_Toc513157134)

[12.2 DEVIATIONS 41](#_Toc513157135)

[13 QUALITY CONTROL AND QUALITY ASSURANCE 41](#_Toc513157136)

[13.1 MONITORING 41](#_Toc513157137)

[13.2 AUDIT 42](#_Toc513157138)

[13.3 STORAGE AND DISCARD OF DATA AND SAMPLES 42](#_Toc513157139)

[13.4 HANDLING IN CASES WHERE SAMPLES AND INFORMATION IS LIKELY TO BE USED FOR FUTURE MEDICAL PRACTICE 42](#_Toc513157140)

[13.5 DIRECT OBSERVATION FOR THE ORIGINAL DOCUMENTS 42](#_Toc513157141)

[14 CONFLICT OF INTEREST AND FUNDING ORGANIZATION 42](#_Toc513157142)

[14.1 CONFLICT OF INTEREST 42](#_Toc513157143)

[14.2 RELATIONSHIP WITH FUNDING ORGANIZATION 43](#_Toc513157144)

[15 COST BURDEN AND COMPENSATION FOR DAMAGE TO PARTICIPANTS’ HEALTH 43](#_Toc513157145)

[15.1 COST BURDEN 43](#_Toc513157146)

[15.2 COMPENSATION FOR DAMAGE TO PARTICIPANTS’ HEALTH 43](#_Toc513157147)

[16 PUBLICATION POLICY AND ATTRIBUTION OF PRODUCTIONS 43](#_Toc513157148)

[16.1 CLINICAL TRIALS REGISTRY 43](#_Toc513157149)

[16.2 PUBLICATION POLICY AND ATTRIBUTION OF PRODUCTIONS 43](#_Toc513157150)

[16.3 ACCESS TO DATA 44](#_Toc513157151)

[17 STUDY COMPLIANCE AND AMENDMENT 44](#_Toc513157152)

[18 STUDY ORGANIZATION 45](#_Toc513157153)

[19 REFERENCES 49](#_Toc513157154)

# 0 PROTOCOL SYNOPSIS

## 0.1 STUDY SCHEME

Figure 1

Infants with atopic dermatitis at the age of 7–13 weeks

N=650

Registration and Randomization

Conventional treatment group, n=325

Standard treatment based on clinical practice guidelines for the management of atopic dermatitis 2016 (1)

(Reactive method)

Aggressive intervention group, n=325

Early aggressive treatment with topical corticosteroids

(Proactive method)

## 0.2 PURPOSE

To test the superiority of aggressive intervention to prevent food allergy over conventional treatment in infantile atopic dermatitis (AD) by a randomized controlled trial (RCT).

## 0.3 STUDY DESIGN

This study is designed as a multicenter, investigator-blinded, randomized, parallel group controlled trial.

## 0.4 PARTICIPANTS

Infants (7–13 weeks old) who develop an itchy rash within the previous 28 days and receive a diagnosis of AD by a dermatologist, based on the U.K. Working Party’s diagnostic criteria

## 0.5 PRIMARY ENDPOINT

Presence of oral food challenge-proven IgE-mediated hen’s egg allergy at 28 weeks of age

## 0.6 STUDY PROCEDURE

**0.6.1 Aggressive intervention group: Early aggressive treatments with topical corticosteroids**

**(Proactive method)**

Basic whole-body treatment

Participants will be followed as described below and administered basic whole-body treatment, except for scalp.

Emollients

|  | Whole body except scalp |
| --- | --- |
| Registration day (Day 0) of the study  to 28 weeks of age | Hirudoid^®^ Soft ointment  every day  twice a day |

Topical corticosteroids

|  | Face | Body except scalp and face |
| --- | --- | --- |
| Registration day (Day 0) to Day 14 of the study | Almeta^®^ ointment  every day  twice a day | Rinderon^®^-V ointment  every day  twice a day |
| Day 15 of the study  to 28 weeks of age | Almeta^®^ ointment  two days per week  twice a day | Rinderon^®^-V ointment  two days per week  twice a day |

Additional skin rash treatment

Participants are to apply TCSs as additional treatment as described below:

| Face | Body except scalp and face | Scalp |
| --- | --- | --- |
| Day 15 of the study  to 28 weeks of age | Day 15 of the study  to 28 weeks of age | Day 15 of the study  to 28 weeks of age |
| Almeta^®^ ointment  every day until rash remission  twice daily | Rinderon^®^-V ointment  every day until rash remission  twice a day | Rinderon^®^-V lotion until rash remission  twice a day |

**0.6.2 Conventional treatment group: Standard treatment based on the Guidelines for the Management of Atopic Dermatitis (2016) (1) (Step-up reactive method)**

Basic whole-body treatment

Participants will be followed as described below and administered basic whole-body treatment, except for scalp.

|  | Whole body except scalp |
| --- | --- |
| Registration day (Day 0) of the study  to 28 weeks of age | Hirudoid^®^ Soft ointment  every day  twice a day |

Additional skin rash treatment

Participants are to apply TCSs as additional treatment as described below:

| Area | Face | | Body except scalp and face | | | Scalp |
| --- | --- | --- | --- | --- | --- | --- |
| Severity of skin rash | Less mild | Mild, moderate, and severe | Less mild | Mild and moderate | Severe | Any severity |
| Registration day (Day 0) of the study  to 28 weeks of age | Without additional treatment | Almeta^®^ ointment  every day until rash remission  once a day | Without additional treatment | Almeta^®^ ointment  every day until rash remission  once a day | Rinderon^®^-V ointment  every day until rash remission  once a day | Rinderon^®^-V lotion until rash remission  once a day |

## 0.7 ANTICIPATED NUMBER OF PARTICIPANTS AND STUDY DURATION

1. The anticipated number of participants is 650: Aggressive intervention group, n=325 and conventional treatment group, n=325.
2. Duration of the study registration will be 2 years after IRB approval.
3. Duration of the study intervention will be 3 years after IRB approval.

## 0.8 CONTACT INFORMATION

Contact from participants, inclusion criteria, patient list-up, adverse events, change of intervention, clinical judgments, and so on: Study-Coordinating Center

Study-Coordinating Center: Division of Allergy, Department of Medical Subspecialties, National Center for Child Health and Development

2-10-1 Okura, Setagaya-ku, Tokyo 157-8535, Japan

Phone: +81-3-3416-0611; Fax: +81-3-3415-9260

E-mail: allergy_research@ncchd.go.jp

Registration, input information on eCRF, etc.: Data Center

Data Center: Division of Data Management, Center for Clinical Research and Development, National Center for Child Health and Development

2-10-1 Okura, Setagaya-ku, Tokyo 157-8535, Japan

Phone: +81-3-3416-0181 (ext: 5083)

E-mail: dcc@ncchd.go.jp

LIST OF ABBREVIATIONS

| ACTH | Adrenocorticotropic Hormone |
| --- | --- |
| AD | Atopic Dermatitis |
| aOR | Adjusted Odds Ratio |
| CI | Confidence Interval |
| CTR | Clinical Trial Registry |
| EASI | Eczema Area and Severity Index |
| EAT Study | Enquiring About Tolerance Study |
| EDC | Electronic Data Capture |
| eCRF | Electronic Case Report Form |
| FA | Food Allergy |
| DFI | Family Impact of Childhood Eczema Questionnaire |
| FTU | Finger-Tip Unit |
| HEAP Study | Hen's Egg Allergy Prevention Study |
| HOME | The global Harmonising Outcome Measures for Eczema |
| IDMC | Independent Data Monitoring Committee |
| IDQoL | Infants’ Dermatitis Quality of Life Questionnaire |
| IgE | Immunoglobulin E |
| IgG | Immunoglobulin G |
| ISAAC | The International Study of Asthma and Allergies in Children |
| IRB | Institutional Review Board |
| ITT | Intention to Treat |
| GCP | Good Clinical Practice |
| LEAP Study | Learning Early About Peanut Allergy Trial |
| NCCHD | National Center for Child Health and Development |
| NICE | National Institute for Health and Clinical Excellence |
| NSAIDs | Non-Steroidal Anti-Inflammatory Drugs |
| OR | Odds Ratio |
| OFC | Oral Food Challenge |
| PACI Study | Prevention of Allergy via Cutaneous Intervention Study |
| PETIT | Prevention of Egg Allergy with Tiny Amount Intake Trial |
| POEM | Patient-Oriented Eczema Measure |
| PP | Per Protocol |
| RCT | Randomized Controlled Trial |
| SPT | Skin Prick Test |
| QoL | Quality of Life |
| SCORAD | SCORing Atopic Dermatitis |
| STEP | Starting Time of Egg Protein Trial |
| TARC | Thymus and Activation-Regulated Chemokine |
| TCSs | Topical Corticosteroids |
| T-CHILD | Tokyo Children's Health, Illness and Development study |
| UK | United Kingdom |
| US | United States of America |
| UMIN-CTR | University Hospital Medical Information Network |

# 1 BACKGROUND

## 1.1 ATOPIC DERMATITIS

1.1.1 ATOPIC DERMATITIS

AD is a disease with repeated exacerbation and remission, that is chiefly characterized by eczema with itch, and it is mostly exhibited by patients with atopic diathesis (personal or family history of bronchial asthma, allergic rhinitis, and conjunctivitis, and/or AD and/or predisposition to overproduction of immunoglobulin E (IgE) antibodies) **(1). Severely affected children have severe sleep disturbance and the condition places a heavy burden on daily life. Epidermal barrier dysfunction plays an important role in AD pathophysiology.** The skin barrier consists of tight junctions of the stratum layer, filaggrin protein with keratin filaments of keratinocytes, desmosomes connecting keratinocytes, natural moisturizing factors with water holding water intracellular lipids with ceramides, sebaceous membranes of outlayer and so on. Filaggrin partially forms the epidermal barrier and filaggrin loss-of-function mutations were found to be more common among patients with AD compared with unaffected populations (2, 3). Reduced filaggrin expression was also found in patients with AD who were not carriers of known filaggrin mutations (4). Two randomized controlled trials (RCTs) were performed to investigate whether protecting the skin barrier with a moisturizer applied at the beginning of the neonatal period would prevent development of infantile AD. These studies demonstrated that application of a moisturizer during early life reduced the incidence of AD/eczema in infants (5, 6). Inflammation on the skin and allergic sensitization is also suggested as a mechanism of AD. Once skin barrier dysfunction occurs, allergens and bacterial toxins from outside the skin penetrate the skin barrier. Langerhans cells extend dendrites through the tight junctions and capture antigens from outside the tight junction barrier (7, 8). It was suggested that T-helper type 2 cells shift allergic sensitization in patients with AD, which might be initiated by the external antigen uptake by Langerhans cells in an inflamed epidermal layer. Various immune cells such as sensitized T cells release cytokines that cause allergic inflammation of the skin. Skin inflammation induces itch on the skin and the itch-scratch cycle reduces skin barrier function.

1.1.2 ATOPIC DERMATITIS EPIDEMIOLOGY

A systematic review of international trends in AD suggested that the prevalence of atopic eczema is increasing in Africa, eastern Asia, western Europe, and parts of northern Europe (e.g., the UK) (9). According to a Japanese national survey, the prevalence of infants at the age of 6 months with AD or a suspected history of AD was about 25% (10). Rudikoff *et al.* reported that onset of AD is within the first year of life in 60% of patients (11). A cohort study in Stockholm demonstrated that children with single or double parental atopic history developed AD with 37.9% or 50.0%, respectively, at 4 years of age (12) and most children with AD have a family history of atopy. The allergic march concerns the development of AD and concomitant sensitization to food and aeroallergens in early childhood, progressing to asthma and allergic rhinitis in later childhood or adult life (13). Of 1-year-old children who ever received a diagnosis of AD, 19.3% had a food allergy (FA) at 3 years of age in the T-CHILD birth cohort study in Japan (14).

1.1.3 ATOPIC DERMATITIS MANAGEMENT

According to the Japanese guidelines for AD (1), there are recommendations for basic treatments, namely, investigate and treat causes and exacerbating factors, correct skin dysfunctions (skin care), and use pharmacotherapy; this is similar to guidelines in other countries.

<Investigate and treat causes and exacerbating factors>

Although the exacerbating factors of AD are different among patients because of age and life environment, patients are encouraged to eliminate factors such as the effects from sweat, house dust mites, pets, *Staphylococcus aureus*, and dryness. Patients are encouraged to control the home environment, reducing these exacerbating factors.

<Skin care>

Skin care is needed to eliminate exacerbating factors such as *S. aureus*, sweat, irritants, and allergens and keep the skin clean. In addition, emollients are applied to maintain the skin barrier function.

<Pharmacotherapy>

Topical corticosteroids (TCSs) and tacrolimus ointments are the mainstream of pharmacotherapy to suppress skin inflammation associated with AD. Tacrolimus has been officially approved for patients 2 years of age and older in Japan.

TOPICAL CORTICOSTEROIDS (TCSs)

TCSs are medications to control inflammation on the skin and are the most frequently used as pharmacotherapy for AD. In Japan, TCS potency has five categories: strongest, very strong, strong, medium, and weak (Table 1). However, TCS potency is not the same in every country. TCS potency in the US has seven categories (15) and that in the UK has four categories (16). The potency of mometasone furoate, betamethasone valerate, and alclometasone dipropionate in Japan, the US, and the UK is summarized in Table 2. For applying TCSs, the finger-tip unit (FTU) is used as an index of the external dose. One FTU is a dose equivalent to approximately 0.5 g of ointment, which can cover the area of two palms of an adult (2% of the body surface area) (17). The approach for using TCSs in AD is either reactive or proactive management (Figure 2) (18, 19). Reactive management is when TCSs are administered only when the rash worsens. Proactive management is applying anti-inflammatory TCS therapy intermittently even after the skin clears to suppress subclinical inflammation of the skin in AD patients. Proactive management is preferable for patients with moderate or severe AD, because reactive management of flare-ups is not always successful to control AD symptoms. An RCT by Hanifin *et al.* compared proactive management using fluticasone propionate cream 0.05% (twice per week) with its vehicle base to reduce the risk of relapse in subjects aged 3 months to 65 years with stabilized AD (20). Pediatric patients were 8.1 times less likely to have an AD relapse (95%CI: 4.3, 15.2; *p*<0.001) and proactive management was considered to reduce the risk of flare-ups. Fukuie *et al.* conducted an RCT to investigate whether, when compared with reactive therapy, 1-year proactive therapy would stabilize AD symptoms (21). They measured serum TARC as a biomarker of AD severity and prevented subsequent sensitization to aeroallergens in children aged 3 to 90 months with AD. At the study end, the SCORAD index was significantly lower in the proactive treatment group compared with the reactive treatment group. In addition, Fukuie *et al.* performed a 2-year retrospective cohort study of patients with moderate to severe AD to investigate whether proactive management changes serum IgE level compared to reactive management (22). Serum total IgE titer was significantly decreased in the proactive treatment group compared with the reactive treatment group (2442 IU/mL vs. 2081 IU/mL; *p*<0.01). In addition, the serum egg white-specific IgE level decreased significantly during follow-up (60.0 IU/mL vs. 36.6 IU/mL; *p*=0.004). In a case control study, infants with early proactive management (beginning at ≤4 months old) had a lower prevalence of egg allergy compared to infants with later proactive management (beginning at ≥5 months) at the age of 18 months (9.1% vs. 24.2%) (23). Previously published results suggest that proactive management might be effective for reducing eczema flare-ups, and for preventing development of allergic sensitization. Thus, early proactive management for infantile AD may reduce the risk of FA.

SAFETY OF TOPICAL CORTICOSTEROIDS

TCSs are topical anti-inflammatory agents and they are safer than systemic steroids administered via an oral or intravenous route. Absorption of TCS in the skin is considered to depend on many factors such as molecular weight of TCS, cream or ointment bases, application amount, potency of TCS, application period, and age. Cutaneous side effects include telangiectasias, skin atrophy, skin striae, focal hypertrichosis, acne-like eruptions, rosacea-like eruptions, folliculitis, and so forth (24, 25). In an observational study in the US, children with moderate or severe AD did not show adrenal suppression although they had used TCSs for more than several years since infancy (26). Fukuie *et al.* performed rapid ACTH stimulation tests for eight children in the proactive group and four children in the reactive group at 3 months after starting the study intervention and found that no children showed adrenal suppression (21). However, Hanifin *et al.* demonstrated that 2/44 children in the proactive group had adrenal suppression in the RCT (20). Children are more susceptible to steroids than adults and, therefore, their treatment should be carefully considered.

Table 1. TCS potency categories in Japan (1)

| Potency | Generic name and strength | Brand name |
| --- | --- | --- |
| Strongest | 0.05% Clobetasol propionate | Dermovate^®^ |
|  | 0.05% Diflorasone diacetate | Diflal^®^, Diacort^®^ |
| Very strong | 0.1% Mometasone furoate | Flumeta^®^ |
|  | 0.05% Betamethasone butyrate propionate | Antebate^®^ |
|  | 0.05% Fluocinonide | Topsym^®^ |
|  | 0.064% Betamethasone dipropionate | Rinderon^®^-DP |
|  | 0.05% Difluprednate | Myser^®^ |
|  | 0.1% Amcinonide | Visderm^®^ |
|  | 0.1% Diflucortolone valerate | Texmeten^®^, Nerisona^®^ |
|  | 0.1% Hydrocortisone butyrate propionate | Pandel^®^ |
| Strong | 0.3% Deprodone propionate | Eclar^®^ |
|  | 0.1% Dexamethasone propionate | Methaderm^®^ |
|  | 0.12% Dexamethasone valerate | Voalla, Zalucs^®^ |
|  | 0.1% Halcinonide | Adcortin^®^ |
|  | 0.12% Betamethasone valerate | Betnevate^®^, Rinderon^®^-V |
|  | 0.025% Fluocinolone acetonide | Flucort^®^ |
| Medium | 0.3% Prednisolone valerate acetate | Lidomex^®^ |
|  | 0.1% Triamcinolone acetonide | Ledercort^®^ |
|  | 0.1% Alclometasone dipropionate | Almeta^®^ |
|  | 0.05% Clobetasone butyrate | Kindavate^®^ |
|  | 0.1% Hydrocortisone butyrate | Locoid^®^ |
| Weak | 0.1% Dexamethasone | Glymesason^®^, Eurason^®^ |
|  | 0.5% Prednisolone | Prednisolone^®^ |

Table 2. Comparison of TCS potency in Japan, the US, and the UK

| TCS | Japan (Ⅰ-Ⅴ) | US (Ⅰ-VII) | UK (Ⅰ-IV) |
| --- | --- | --- | --- |
| Fulumeta^®^  (Mometasone furoate) | Very strong (II) | High (II) | Potent (II) |
| Rinderon^®^-V  (Betamethasone valerate) | Strong (III) | Medium (III) | Potent (II) |
| Almeta^®^  (Alclometasone dipropionate) | Medium (IV) | Low (IV) | Moderate (III) |

Figure 2. Reactive and proactive management plan for AD (19)

## 1.2 FOOD ALLERGY

1.2.1 FOOD ALLERGY

Food allergy (FA) is defined as “a phenomenon in which adverse reactions are caused through antigen-specific immunological mechanisms after exposure to a given food” (27). FA is classified into two categories, namely, IgE-mediated FA and non-IgE mediated FA. IgE-mediated FA, which is more common than non-IgE mediated FA, causes allergic reactions within 2 hours and may cause anaphylaxis, which is life-threatening. Patients with IgE-mediated FA are sensitized to causal food, resulting in an elevation of food allergen-specific IgE titers. Although all patients who are sensitized to food allergens do not necessarily develop to adverse reactions to food allergens, the higher the IgE levels, the higher the risk of anaphylaxis. Patients with FA often have a lower quality of life as a result of restrictions on eating causal food, which is required because of their high risk of developing anaphylaxis (28).

1.2.2 FOOD ALLERGY EPIDEMIOLOGY

The prevalence of FA has been increasing worldwide (29). In Japan the most common causal allergen to FA was hen’s egg and the second most was cow’s milk (30). A hospital-based cohort study in Tokyo reported that the cumulative incidence of FA and hen’s egg allergy was 9% and 5.1%, respectively, among children aged 12 months (14). The prevalence of FA among children in elementary school has been increasing according to a national survey in Japan. The systematic review and meta-analysis demonstrated that cow’s milk and hen’s egg allergies were more common among younger children and the prevalence of food challenge-defined allergy to hen’s egg among 0–1-year-olds was 0.75% (0.46–1.04) among European populations (31). According to Table 3, the results of the prevalence of hen’s egg allergy showed large variations. The reason is considered to be that there are differences in diagnostic method for FA, definition, age, area, patients background, and so on.

Table 3. Observational studies of hen’s egg allergy and hen’s egg sensitization among infants

| Year, country, study design | Hen’s egg allergy | | | Hen’s egg sensitization | | |
| --- | --- | --- | --- | --- | --- | --- |
|  | All infants | With  eczema | Without  eczema | All infants | With  eczema | Without  eczema |
| 2007, Australia, cross-sectional (32) |  |  |  |  | 73% (34 w, 20-51 w) SPT  80% (34 w, 20-51 w) IgE |  |
| 2008, China, cross-sectional (33) |  |  |  |  | 70% (≤12 m) SPT |  |
| 2011, Sweden, cohort (34) |  |  |  | 24.8% (6 m) sIgE  32% (12 m) sIgE  27% (24 m) sIgE  51% (60 m) sIgE |  |  |
| 2011, Australia, cohort (35) | 8.9% (12 m) OFC |  |  | 16.5% (12 m) SPT≥1 mm  11.7% (12 m) SPT≥3 mm |  |  |
| 2012, Japan, cohort (36) |  |  |  |  | 87.5% (14 m) sIgE | 31.9% (14 m) sIgE |
| 2012, Iran, cross-sectional (37) |  |  |  |  | 17.7% (2-48 m) SPT |  |
| 2015, Australia, cohort (38) |  | 18.8% (12 m) OFC | 3.7% (12 m) OFC |  | 24.1% (12 m) SPT≥2 mm or sIgE≥0.35 | 5.2% (12 m) SPT≥2 mm or sIgE≥0.35 |
| 2016, Japan, cohort (14) | 5.2% (12 m)  questionnaire |  |  |  |  |  |

OFC, oral food challenge; SPT, skin prick test; sIgE, specific IgE titers; w, weeks of age; m, months of age

1.2.3 ATOPIC DERMATITIS AND SENSITIZATION THROUGH THE SKIN

Lack *et al.* demonstrated that peanut allergy was positively associated with the use of skin care products containing peanut oil in England (39). The results from the study suggested that peanut allergens absorbed through the skin may cause allergic sensitization. Van den Oord *et al.* demonstrated, in a systematic review and meta-analysis, that filaggrin gene defects among children increased the risk of developing allergic sensitization (OR 1.57; 95%CI: 1.20, 2.07) (40). Flohr *et al.* also showed that young infants with AD had an increased risk of allergic sensitization at the age of 3 months (aOR 6.18; 95%CI: 2.94, 12.98; *p*<0.001) (41). Spergel *et al.* showed that total serum IgE and serum egg white-specific IgE in a cohort study increased in patients with increased AD severity (42). From a review of past studies (Table 3), the prevalence of hen’s egg allergy or hen’s egg sensitization in infants varies depending on various characteristics such as with/without eczema, timing of hen’s egg introduction, country, age, and diagnostic method for FA and infants with eczema tend to have higher prevalence of IgE sensitization than those without eczema.

1.2.4 ATOPIC DERMATITIS AND FOOD ALLERGY

Martin *et al.* reported that 20% of 1-year-old infants who had a history of eczema received a diagnosis of FA (38). Additionally, 50.8% of infants (95%CI: 42.8, 58.9) with early eczema onset (<3 months old) who required doctor-prescribed topical corticosteroid treatment developed challenge-proven FA. Shoda *et al.* demonstrated that in each age (by month) stratum, infants with onset of eczema within the first 1–2 months after birth had the highest risk of FA at 3 years of age (aOR 6.61; 95%CI: 3.27, 13.34; *p*<0.001) and the second highest was within 3–4 months after birth (aOR 4.69; 95%CI: 2.17, 10.13) (14). The results from previous studies suggest that infants who develop AD in early infancy have a higher risk for FA. We summarize the prevalence of hen’s egg sensitization among patients with AD in Table 3.

1.2.5 ORAL TOLERANCE

Oral tolerance is defined as a state of suppression or non-reactivity towards immunological response to oral allergen exposures from eating the allergenic food. The gastrointestinal mucosa is exposed to a wide variety of antigens, but most of food-derived antigens are not harmful to humans. This is because humans have an antigen recognition mechanism for inducing immune tolerance, not for providing unnecessary immune response against food antigens (27). However, Du Toit *et al.* reported that Jewish children in the UK had a 10-fold higher prevalence of peanut allergy than Jewish children in Israel (43). The major explanation was that there is a difference in dietary habits between the UK and Israel. It was suggested that the possibility that the parents of children in Israel do not restrict intake of peanuts during infancy, whereas parents in the UK limit intake of peanuts during infancy (Du Toit *et al.*). Du Toit and colleagues conducted a randomized controlled trial (LEAP Study) to examine the prevention of peanut allergy by early peanut ingestion in 640 infants with atopic dermatitis and egg allergy (44). Infants in the consumption group who initially had negative results on the skin-prick test started taking peanuts when they were at least 4 months old and less than 11 months old at randomization. The prevalence of peanut allergy in the avoidance group at 60 months of age was significantly higher than that in the consumption group (13.7% vs. 1.9%; *p*<0.001). The findings supported the past results of epidemiological studies in Jews.

Perkin *et al.* conducted an RCT (EAT Study) that examined whether infants in the general population can prevent the onset of food allergy at 3 years old by regularly consuming allergenic foods such as hen’s eggs from 3–4 months after birth. Regarding hen’s eggs, the early consumption group (n=569) was to take ½ of a heated hen’s egg twice a week from 3–4 months after birth periodically according to the study, but only 215 participants could take heated hen’s egg according to the study protocol. The prevalence of hen’s egg allergy was 3.7% in the early consumption group (n=569) and 5.4% in the standard consumption group (n=596) by the Intention to Treat (ITT) analysis, and 1.4% in the early consumption group (n=215) and 5.5% in the standard consumption group (n=525) by the Per Protocol (PP) analysis. There was no significant difference in the prevalence of hen’s egg allergy between both groups in the ITT analysis (45). Bellach *et al.* conducted an RCT (HEAP Study) to investigate the efficacy and safety of early hen’s egg introduction at age 4–6 months to prevent hen’s egg allergy in the general population (46). The early consumption group took egg white powder (equivalent to ⅓ of a hen’s egg) three times per week from 4–6 months old to 12 months, while the placebo group eliminated hen’s egg and took rice flour three times per week from 4–6 months old to 12 months. The prevalence of egg allergy among the participants at 12 months of age in the HEAP Study was 2.1% in the early consumption group (n=142) and 0.6% in the placebo group (n=156) (relative risk 3.30; 95%CI: 0.35, 31.32).

Palmer *et al.* also performed an RCT (STEP Study) to examine if regular egg protein consumption from 4–6 months of age reduces a risk of IgE-mediated hen’s egg allergy in high-risk infants without eczema (47). The early consumption group took 0.9 g of pasteurized raw hen’s egg powder every day from 4-6 months old to 10 months after birth (equivalent to ½ of a whole egg in one week), and the placebo group took powder without hen’s egg every day from 4-6 months old to 10 months and eliminated hen’s egg. The prevalence of IgE-mediated hen’s egg allergy at the age of 12 months was 7.0% in the early consumption group (n=407) and 10.3% in the placebo group (n=413) (adjusted relative risk 0.75; 95%CI: 0.48, 1.17), but did not show a statistically significant difference. On the other hand, Natsume *et al.* conducted a double-blind, placebo-controlled RCT (PETIT Study) for infants 4–5 months of age with eczema who were enrolled and randomly assigned to the early introduction of egg or placebo to examine the proportion of participants with hen’s egg allergy confirmed by open oral food challenges at 12 months of age (48). Participants in the early egg consumption group consumed 0.2 g of heated hen’s egg every day during 6–8 months of age and 1.1 g of heated hen’s egg every day during 9–12 months of age. The participants’ eczema was aggressively treated at entry and skin conditions of most participants were maintained without exacerbations throughout the intervention period. In the study, 121 participants revealed that the prevalence of egg allergy was 37.7% in the placebo group (n=61) and 8.3% in the egg group (n=60), and the risk ratio was 0.221 (95%CI: 0.090, 0.543; *p*=0.00013); this demonstrated that the prevalence in the egg group was significantly lower than that in the placebo group. The results among these three RCTs were different and the reasons were considered to be different background of participants (high risk infants, or general infants), race, method of egg consumption, eczema control level, and so on. Immune tolerance is not considered to be induced merely by orally taking allergenic foods from the early stage of infancy.

1.2.6 Dual-allergen-exposure hypothesis

Lack *et al.* suggested the dual-allergen-exposure hypothesis which states that tolerance is induced from oral exposure to food antigen, enhancing immune response to suppress allergy and that infants with eczema are exposed to food antigen via skin and induce immune cells, enhancing allergy and producing IgE antibodies (sensitization) (Fig. 3) (49). This hypothesis implies that it is important not only to induce oral immune tolerance, but to prevent allergic sensitization through the skin to reduce allergies.

Figure 3. Dual-allergen-exposure hypothesis (49)

1.2.7 TREATMENT FOR FOOD ALLERGY

FA treatment is basically the minimum necessary food elimination based on correct diagnosis. We should ensure safety (50). In the level of clinical studies, several clinical trials have been conducted to evaluate the efficacy and safety of immunotherapy, inducing immune tolerance (51). Caminiti *et al.* performed an RCT regarding oral immunotherapy of hen’s egg for children with hen’s egg allergy (52). The patients who received 4-month oral immunotherapy (n=16/17) were desensitized to hen’s egg compared to patients in the placebo group who were not desensitized (n=0/14). However, when OFC of hen’s egg was conducted after elimination of hen’s egg for 3 months after continuing taking hen’s egg for 10 months from the initial intervention, 31% (5/17 people) showed symptoms for hen’s egg allergy again. It was suggested that food allergy reactions might be induced for hen’s egg if hen’s egg is eliminated for a long period of time. Oral immunotherapy for food allergy is still at the research level and is not recommended as clinical practice in the food allergy guidelines not only in Japan, but also in Europe and the United States. In the future, development of oral immunotherapy as a treatment for food allergy will be expected to be available to induce immune tolerance for causal food allergen and to be applied to daily clinical practice.

## 1.3 EXPECTATIONS FOR EARLY AGGRESSIVE INTERVENTION FOR ATOPIC DERMATITIS

Allergic march refers to the natural history or typical progression of allergic diseases that often begin early in life. These include AD (eczema), FA, allergic rhinitis (hay fever), and asthma (53). In most patients, AD is the first clinical manifestation with the highest incidence in the first year of life and those affected develop other allergic diseases such as FA, asthma, and allergic rhinitis later in childhood. We hypothesize that to prevent future allergic march, an appropriate intervention for AD, which emerges at the first stage of allergic march, is important to be considered. We expect that early aggressive intervention for AD will likely prevent development of later allergen sensitization, FA, asthma, and allergic rhinitis.

## 1.4 NECESSITY AND SIGNIFICANCE OF THE STUDY

Shoda *et al.* demonstrated that in each age (by month) stratum, infants with onset of eczema within the first 1–2 months after birth had the highest risk of FA at 3 years of age (aOR 6.61; 95%CI: 3.27, 13.34; *p*<0.001) and the second highest was within 3–4 months after birth (aOR 4.69; 95%CI: 2.17, 10.13) (14). Infants who develop AD in early infancy have a high risk of developing FA. The increasing prevalence of FA is a current worldwide epidemic. According to the national survey by the ministry of education, culture, sports, science and technology, the percentage of school children with past history of anaphylaxis was 0.14% in 2004 and 0.5% in 2013, an increase of nearly 4 times (54). As we mentioned before, we think that it is important to introduce oral immune tolerance by early hen’s egg consumption and prevent sensitization via skin to prevent the development of food allergy. To our knowledge, this would be the first RCT study to examine the efficacy of early aggressive intervention for AD to prevent later FA. If we establish a novel new strategy for prevention of FA development by early aggressive intervention for AD, it is expected that quality of life in patients will improve, the prevalence of FA will decrease, and the medical care cost will be reduced.

# 2 PURPOSE

The purpose is to test by a randomized controlled trial the superiority of aggressive intervention over conventional treatment of infantile atopic dermatitis (AD) to prevent food allergy.

## 2.1 STUDY HYPHOTHESIS

Aggressive intervention is superior to conventional treatment to prevent the development of IgE-mediated hen’s egg allergy.

# 3 DEFINITIONS

**Atopic dermatitis (AD)** In this study, we use the U.K. Working Party’s diagnostic criteria for AD (55-57). The U.K. Working Party’s diagnostic criteria were validated as diagnostic criteria for AD by Williams *et al.* AD is a diagnosis with one major criterion plus three or more minor criteria in the U.K. Working Party’s diagnostic criteria (Table 4).

Table 4. The U.K. Working Party’s diagnostic criteria

| Major criterion |
| --- |
| An itchy skin condition (or parental report of scratching or rubbing in a child) |
| Minor criteria |
| History of involvement of the skin creases such as folds of elbows, behind the knees, front of ankles, or around the neck (including cheeks in children under 10). |
| A personal history of asthma or hay fever (or history of atopic disease in a first-degree relative in children under 4). |
| A personal history of general dry skin in the last 12 months. |
| Visible flexural eczema (or eczema involving the cheeks/forehead and outer limbs in children under 4. |
| Onset under the age of 2 (not used if child is under 4). |

**Skin rash severity** The definition of severity for skin rash is taken from the clinical practice Guidelines for the Management of Atopic Dermatitis (2016) (1), which are listed below (Table 5).

Table 5. Skin rash severity

| Skin rash severity | Cutaneous manifestations |
| --- | --- |
| Less mild | Primarily dryness with negligible inflammation |
| Mild | Primarily dryness, mild erythema and scales |
| Moderate | Primarily moderate erythema, scales, a few papules and excoriations |
| Severe | Primarily severe swelling/edema/infiltration or erythema with lichenification, multiple papules, severe scales, crusts, vesicles, erosion, multiple excoriations and pruriginous nodules |

**Disease-free day** Condition without skin rash caused by AD on any area of the body.

**Degree of rescue treatment for atopic dermatitis during the study**

The amount of applied Fulumeta^®^ ointment is used as the evaluation of the degree of rescue treatment.

**Infants’ Dermatitis Quality of Life Questionnaire (IDQoL)**

IDQoL was developed by Lewis-Jones *et al*. as an evaluation scale of QoL for infants under 4 years old with atopic dermatitis (58, 59).

**Family Impact of Childhood Eczema Questionnaire (DFI)**

DFI was developed by Lawson *et al*. as an evaluation scale of QoL for the family members who have a child with atopic

dermatitis (60). Parents answer the questionnaire.

**EASI (Eczema Area and Severity Index)** The EASI score, developed by Hanifin *et al.*, is a tool used to measure the extent (area) and severity of atopic eczema (Eczema Area and Severity Index) (61). In this study, a medical doctor evaluates the eczema region, extent (area) and severity of eczema, and calculates an EASI score. EASI has been recommended as an outcome measure for AD in clinical trials by the global Harmonising Outcome Measures for Eczema (HOME) (62).

**POEM (Patient Oriented Eczema Measure)** The POEM score, developed in 2004 by Williams *et al*., is a tool used to measure severity of AD by patients (caregivers) (63). The POEM questionnaire consists of 7 questions evaluating skin dryness, skin redness, sleep disturbance, and so forth, due to AD. Caregivers answer the questionnaire once a week and severity of AD is evaluated by the POEM score. POEM has been recommended as an outcome measure for AD by caregivers in the HOME (64).

One finger-tip unit (FTU)

One FTU describes the amount of cream squeezed out of its tube onto the end of the finger, which is almost equal to 0.5 g of cream. One FTU is enough to treat an area of skin twice the size of the flat palmar surface of an adults’ hand with fingers together (17).

**IgE-mediated hen’s egg allergy proven by the oral food challenge test (OFC)**

A positive open hen’s egg challenge test at the age of 28 weeks is assessed by a blinded physician (see 6.2). Following the PRRACTALL criteria in Table 6 (65), the challenge tests are scored as positive if participants meet any of the three criteria below within 2 hours after last ingesting hen’s egg:

1. One or more [A] scoring areas
2. Three or more [B] scoring areas
3. Persistence of one or more [B] scoring areas

Table 6. Scoring the challenge outcome (PRRACTALL criteria) (65)

| Ⅰ.  Skin | A. Erythematous rash: % area involved (see body surface area diagram), using rule of five.  0 = Absent  1 = Mild, few areas of faint erythema / 1-3 spots, 5-10％  2 = Moderate areas of erythema / >10%, ≤50%  3 = Severe generalized marked erythema / >50%  Figure 4. Rule of five  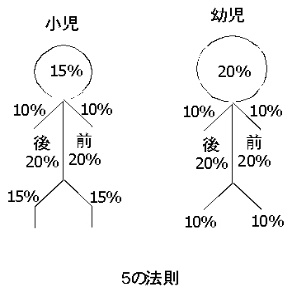 | |
| --- | --- | --- |
|  | B. Pruritus | 0 = Absent |
|  |  | 1 = Mild, occasional scratching |
|  |  | 2 = Moderate: scratching continuously for >2 minutes at a time |
|  |  | 3 = Severe: hard continuous scratching, excoriations [B] |
|  | C. Urticaria / angioedema | 0 = Absent |
|  |  | 1 = Mild: less than 3 hives, or mild lip edema [B] |
|  |  | 2 = Moderate: more than 3 and less than 10 hives, or significant lip or face edema [A] |
|  |  | 3 = Severe: generalized involvement [A] |
|  | D. Rash | 0 = Absent |
|  |  | 1 = Mild: few areas of faint erythema |
|  |  | 2 = Moderate: areas of erythema [B] |
|  |  | 3 = Severe: generalized marked erythema (>50%) [A] |
| Ⅱ. Upper respiratory | A. Sneezing/ Itching | 0 = Absent |
|  |  | 1 = Mild: rare bursts, occasional sniffling |
|  |  | 2 = Moderate: bursts <10, intermittent rubbing of nose and/or eyes or frequent sniffling [B] |
|  |  | 3 = Severe: continuous rubbing of nose and/or eyes, periocular swelling and/or long bursts of sneezing, persistent rhinorrhea [A] |
| Ⅲ.  Lower respiratory | A. Wheezing | 0 = Absent |
|  |  | 1 = Mild: expiratory wheezing on auscultation [A] |
|  |  | 2 = Moderate: inspiratory and expiratory wheezing [A] |
|  |  | 3 = Severe: use of accessory muscles, audible wheezing [A] |
|  | B. Laryngeal | 0 = Absent |
|  |  | 1 = Mild: >3 discrete episodes of throat clearing or cough, or persistent throat tightness/pain [B] |
|  |  | 2 = Moderate: hoarseness, frequent dry cough [A] |
|  |  | 3 = Severe: stridor [A] |
| Ⅳ.  Gastrointestinal | A. Subjective Complaints | 0 = Absent |
|  |  | 1 = Mild: complaints of nausea or abdominal pain, itchy mouth/throat [B] |
|  |  | 2 = Moderate: frequent complaints of nausea or pain with normal activity [B] |
|  |  | 3 = Severe: notably distressed due to GI symptoms with decreased activity [B] |
|  | B. Objective Complaints | 0 = Absent |
|  |  | 1 = Mild: 1 episode of emesis or diarrhea [B] |
|  |  | 2 = Moderate: 2-3 episodes of emesis or diarrhea or 1 of each [A] |
|  |  | 3 = Severe: >3 episodes of emesis or diarrhea or 2 of each [A] |
| Ⅴ.  Cardiovascular |  | 0 = Absent: normal heart rate and/or blood pressure for age or patient’s baseline |
|  |  | 1 = Mild: subjective response (weak, dizzy), or tachycardia [B] |
|  |  | 2 = Moderate: drop in blood pressure and/or >20% from baseline, or significant change in mental status [A] |
|  |  | 3 = Severe: cardiovascular collapse, signs of impaired circulation (unconsciousness) [A] |

**IgE-mediated FA proven by medical interview**

IgE-mediated FA is determined by study physicians’ interviews. IgE-mediated FA is considered positive if participants have a history of immediate adverse reactions to causal food within 2 hours after ingesting it during the study.

**Wheezing proven by medical interview**

Wheezing is defined if participants have a history of wheezing and determined by study physicians’ interviews during the study.

**Sensitization**

Sensitization is defined as a serum specific IgE titer of 0.35 UA/mL and over.

**Adherence index** An adherence index is determined by the percentage of TCSs applied in accordance with the protocol (days of actual TCS application/days of TCS application required in the study protocol). Good adherence is defined as an adherence index of 70% or more. Poor adherence is defined as an adherence index of less than 70%.

**Age in days and age in weeks** Birth day is defined as an age of 0 days. Age in weeks is defined as listed below.

| Age in weeks | | Age in days | | |  |
| --- | --- | --- | --- | --- | --- |
| 1 | week | 0 | – | 6 | days |
| 2 | weeks | 7 | – | 13 | days |
| 3 | weeks | 14 | – | 20 | days |
| 4 | weeks | 21 | – | 27 | days |
| 5 | weeks | 28 | – | 34 | days |
| 6 | weeks | 35 | – | 41 | days |
| 7 | weeks | 42 | – | 48 | days |
| 8 | weeks | 49 | – | 55 | days |
| 9 | weeks | 56 | – | 62 | days |
| 10 | weeks | 63 | – | 69 | days |
| 11 | weeks | 70 | – | 76 | days |
| 12 | weeks | 77 | – | 83 | days |
| 13 | weeks | 84 | – | 90 | days |
| 14 | weeks | 91 | – | 97 | days |
| 15 | weeks | 98 | – | 104 | days |
| 16 | weeks | 105 | – | 111 | days |
| 17 | weeks | 112 | – | 118 | days |
| 18 | weeks | 119 | – | 125 | days |
| 19 | weeks | 126 | – | 132 | days |
| 20 | weeks | 133 | – | 139 | days |
| 21 | weeks | 140 | – | 146 | days |
| 22 | weeks | 147 | – | 153 | days |
| 23 | weeks | 154 | – | 160 | days |
| 24 | weeks | 161 | – | 167 | days |
| 25 | weeks | 168 | – | 174 | days |
| 26 | weeks | 175 | – | 181 | days |
| 27 | weeks | 182 | – | 188 | days |
| 28 | weeks | 189 | – | 195 | days |

**Nutrition** Nutrition methods are defined as follows:

Breastfeeding: ingesting maternal breast milk

Regular cow’s milk formula feeding: ingesting regular cow’s milk formula

Hypoallergenic infant formula feeding: ingesting partially hydrolyzed and extensively hydrolyzed formulas

Mixed feeding: ingesting by both breastfeeding and infant formula feeding

Weaning food: ingesting semi-solid and/or solid foods

# 4 ELIGIBILITY

Infants with AD who meet all of the following inclusion criteria and none of the exclusion criteria

## 4.1 INCLUSION CRITERIA

Infants (7–13 weeks old) who develop an itchy rash within the previous 28 days and receive a diagnosis of AD based on the U.K. Working Party’s diagnostic criteria

Justification for inclusion criteria

All infants with AD, including those with a family history of AD, will be included. The reason is that we would like to apply the study results for all infants with AD, not only for infants with a specific family background. As the U.K. Working Party’s diagnostic criteria is validated and has been widely used in various intervention studies internationally (66), we will apply the criteria in our study as well. Martin *et al.* reported that 20% of infants at 1 year of age who had a history of eczema received a diagnosis of FA (38). There were 50.8% of infants (95%CI: 42.8, 58.9) with early eczema onset (<3 months) who required doctor-prescribed topical corticosteroid treatment who developed challenge-proven FA. Shoda *et al.* demonstrated that in each age stratum (by month), infants with onset of eczema within the first 1–2 months after birth had the highest risk of FA at 3 years of age (aOR 6.61; 95%CI: 3.27, 13.34; *p*<0.001) (14). Infants who develop AD in early infancy are considered to have a higher risk for later FA and the defined target population in the PACI study is infants at the age of 7–13 weeks. We will exclude infants less than 6 weeks of age because differential diagnosis is difficult between AD and other skin diseases such as seborrheic dermatitis. Infants with AD at the age of 7 weeks and over are target populations because AD diagnosis is available.

## 4.2 EXCLUSION CRITERIA

1. Infants born before 37 weeks of gestation
2. Twin or multiple
3. History of emollient (heparinoid cream: Hirudoid^®^ Soft ointment) and TCSs (alclometasone dipropionate: Almeta^®^; betamethasone valerate: Rinderon^®^-V; mometasone furoate: Fulumeta^®^) side effects
4. History of taking oral or intravenous steroids within the previous 28 days
5. History of taking immunosuppressive agents (cyclosporine, tacrolimus, and so forth) or biologics, except vaccinations or intravenous immunoglobulins, within the previous 28 days
6. IgE-mediated hen’s egg allergy
7. Infants whose immediate family plans to move and who may not be able to visit the study site at 28 weeks of age
8. Parents unable to understand Japanese
9. Unwillingness to adhere to the study requirements and procedures
10. Infants with severe disease and other skin diseases that affect dermatological evaluation and study physicians judge that they are not appropriate for study participation

Justification for exclusion criteria

(1) The corrected age should be applied to infants with preterm birth.

(2) Siblings could potentially be treated with study drugs in error.

(3) Participants cannot apply the study drugs.

(4) (5) (6) (7) (8) These criteria affect the evaluation of the primary outcome.

(9) (10) The parents would not provide informed consent.

# 5 STUDY PROCEDURES

## 5.1 STUDY DESIGN

This study is a multicenter, investigator-blinded, randomized, parallel group controlled trial.

5.1.1 **Aggressive intervention group**: Early aggressive treatments with topical corticosteroids (proactive method)

Hanifin *et al.* performed an RCT for participants who were at least 3 months old and who had moderate or severe AD, to examine the efficacy of proactive therapy (21). Early aggressive treatments are modified from the Hanifin *et al.* study intervention, and details are described in section 5.6.1.

5.1.2 **Conventional treatment group**: Standard treatment based on the Guidelines for the Management of Atopic Dermatitis (2016) (1) (Step-up reactive method)

Standard treatment is based on the Guidelines for the Management of Atopic Dermatitis (2016) (1), and the details are described in section 5.6.1.

5.1.3 ANTICIPATED PARTICIPANTS AND STUDY DURATION

Anticipated number of participants: N=650 (aggressive intervention group: n=325 and conventional treatment group: n=325)

Duration of the study registration: 2 years from the day of IRB approval

Duration of the study intervention: 3 years after IRB approval

The study duration will be from study entry until completion of primary outcome assessment or until study termination. During the study duration, study treatment is continued unless the study treatment discontinuance criteria are satisfied. The study duration will be postponed under IRB approval if the study is not completed as scheduled.

Justification for the anticipated number of participants

Palmer *et al.* performed a double-blind, randomized controlled trial for infants with AD to test whether or not one-sixth of a raw whole egg taken every day can prevent development of IgE-mediated egg allergy. The infants were allocated to receive 1 teaspoon of pasteurized raw whole egg powder (n=49) or rice powder (n=37) daily from 4 to 8 months of age (67). A high proportion (31% [15/49]) of infants randomized to receive egg had an allergic reaction to the egg powder and 20% (10/49) had a reaction at the first ingestion of egg powder (one-sixth of a raw whole egg) at 4 months of age. When the control group participants received half of a raw egg provocation test at 12 months, 51% (18/35) were diagnosed with IgE-mediated egg allergy. These data suggested that prevalence of egg allergy in infancy is likely to depend on the provocation dose of egg and the preparation of egg such as heated or raw, and also on age and race of the participant. Therefore, we estimate that at least 30% of the participants in the conventional treatment group in this study will be diagnosed with IgE-mediated hen’s egg allergy using an oral food challenge test at the age of 28 weeks. However, the prevalence of IgE-mediated hen’s egg allergy among infants treated with early aggressive intervention for AD has never been reported. In Japan, it is common that infants at the age of 28 weeks do not eat hen’s eggs, and the prevalence of hen’s egg allergy at the age of 28 weeks in Japan is unclear. We consider a clinically effective prevalence of infants with early aggressive intervention for AD to be 20%. As discussed above, the calculated sample size is 614. Taking into consideration that the rate of drop out is about 5%, we determined the anticipated number to be 650.

EXPECTED PARTICIPANTS

In 2015, about 1,500 babies at the National Center for Child Heath and Development, 500 at Keio University Hospital, and 500 at Hamamatsu University Hospital were born. The total number of newborn babies was 2,500. According to a national survey in Japan, the prevalence of infants at the age of 6 months with AD or suspected history of AD was about 25% (10). From this previous result, we estimate that there will be about 625 newborn babies who are eligible participants. We assume that half of them (n=312) each year will visit one of the investigational sites for this Study because this is an intervention study. Therefore, we will recruit the expected participants in 2 years. In addition, we plan to increase the number of investigational sites.

## 5.2 REGISTRATION

This study uses VIEDOC^TM^ selected as the Electronic Data Capture system (EDC) by **Pharma Consulting Group Japan K.K.** to register and allocate participants.

Registration procedure

1. Prior to registration in this study, patients as candidates are listed on the patient list-up form (Supplement 1). Study physicians confirm eligibility criteria using the candidate confirmation sheet (Supplement 2). Based on the result of the confirmation of eligibility criteria, study physicians obtain informed consent for study participation using a fixed document request from the proxy (parent or legal guardian) of the participants who meet all of the inclusion criteria and none of the exclusion criteria. If a patient is determined to be ineligible, study physicians record the result on the patient list-up form (Supplement 1).
2. Study physicians input patient information to the EDC for the patient with informed consent. The information is checked immediately by the EDC and a participant identification number is issued and study treatment is assigned if eligibility is satisfied.
3. Participant identification number and assignment group are immediately displayed on the EDC and automatically notifies the principle investigator at the study site, study physician, research secretariat, and data center via e-mail. The principle investigator at the study site prints the assignment information and stores it in the case file.
4. The study physician confirms the assignment result and carries out the assigned study treatment.

Notes on registration

1. For participants who meet ineligibility and withdraw consent before registration, information is not entered into the electronic case report (eCRF).
2. When input of patient information is incomplete, registration can not be accepted until all items are input.
3. Eligibility is confirmed on the registration screen, and completion of registration is made as participant identification number is issued.
4. Registration is not cancelled for participants once they are registered.
5. When misregistration or duplicate registration is found, the data center must be promptly contacted using e-mail.
6. When duplicate registration is made, the prior registration is prioritized.

## 5.3 RANDOMIZATION AND STRATIFICATION

Stratified block assignment with the number of weeks after birth (7 to 10 weeks, 11 to 13 weeks) as the factor in the order of registration is performed and participants are randomly assigned to the “aggressive intervention group” or “conventional treatment group” in a ratio of 1:1.

Justification for stratification

At an early onset, atopic dermatitis tends to be severe, so the number of weeks after birth was assigned as an adjustment factor.

## 5.4 BLINDING

Participants, parents, and investigators who treat participants with AD are not blinded to the study intervention. The physicians who perform OFC and examine the skin are blinded to the group allocations. Those who are blinded to the study intervention are required to meet the following criteria.

1. Blinded physicians are not the physicians who treat participants in the PACI Study.
2. Blinded physicians do not look at documents and all other information about the study intervention.

## 5.5 PATIENT BACKGROUND SURVEY AND START OF STUDY TREATMENT

Study physicians examine the participants’ background at study entry. They will prescribe study drugs for participants and start to treat them.

## 5.6 STUDY INTERVENTION

5.6.1 STUDY INTERVENTION FOR BOTH GROUPS

Proxies (parents or legal guardians) of the participants will obtain skin care education and nutritional education for participants on the day of study registration.

**Skin care education**

Skin care education is performed based on the Guidelines for the Management of Atopic Dermatitis (2016) (1). Proxies (parents or legal guardians) of the participants watch an instructional video on how to wash the body. They are given an information sheet.

**Education for applying ointments**

In the aggressive treatment group and the conventional treatment group, the presence of eczema, its severity, and the topical medicines that are applied according to the sites and duration of application are defined as follows (5.6.1, 5.6.2). The applied amount is 1 FTU. The study physician distributes instructions for applying topical medicines and determining the severity of skin rash to the proxies (parents or legal guardians) or their families at the time of registration so that participants can be treated with study medications at home. The study physician prescribes the topical medicines to be used, and the proxy (parents or legal guardians) or their families conduct the study treatment using the prescribed topical medicines. The proxy (parents or legal guardians) or their families do not discard the topical medicines that are not used, and bring all containers and all remaining topical medicines at the time of visit. Study physicians and/or staff instruct the proxies (parents or legal guardians) or their families to record the application situation of topical medications and severity of the skin rash in the treatment diary, and confirm the log of the treatment diary for each visit.

**Nutritional education**

Study physicians provide information about nutrition at the entry. It is recommended to introduce solid food to infants at 4–5 months of age. Breastfeeding is encouraged to continue until at least 6 months of age. Mothers who are breastfeeding are not restricted from ingesting hen’s egg. Participants are not permitted to eat hen’s egg until the oral food challenge test at the age of 28 weeks is complete. The nutritional intake status of participants is checked at the visit. In this study, unapproved medications and medical devices are not used.

5.6.2 STUDY TOPICAL MEDICINES

Participants apply the emollients and TCSs described below as study drugs.

Emollients: Heparinoid cream (Hirudoid^®^ Soft ointment)

TCSs: Alclometasone dipropionate (Almeta^®^), betamethasone valerate (Rinderon^®^-V), and mometasone furoate (Fulumeta^®^)

Study physicians obtain the most recent information about the study medicines (Hirudoid^®^ Soft ointment, Almeta^®^, Rinderon^®^-V, and Fulumeta^®^) from their package inserts.

5.6.3 Aggressive intervention group: Early aggressive treatments with topical corticosteroids

(Proactive method)

Basic whole-body treatment

Participants will be followed as described below and administered basic whole-body treatment, except for scalp.

Emollients

|  | Whole body except scalp |
| --- | --- |
| Registration day (Day 0) of the study  to 28 weeks of age | Hirudoid^®^ Soft ointment  every day  twice a day |

Topical corticosteroids

|  | Face | Body except scalp and face |
| --- | --- | --- |
| Registration day (Day 0) to Day 14 of the study | Almeta^®^ ointment  every day  twice a day | Rinderon^®^-V ointment  every day  twice a day |
| Day 15 of the study  to 28 weeks of age | Almeta^®^ ointment  two days per week  twice a day | Rinderon^®^-V ointment  two days per week  twice a day |

If participants do not have remission of atopic dermatitis on the 14th day (visit 2) after registration, daily administration of topical corticosteroid steroids is continued until day 28 (visit 3) after registration.

Additional skin rash treatment

Participants are to apply TCSs as additional treatment as described below:

| Face | Body except scalp and face | Scalp |
| --- | --- | --- |
| Day 15 of the study  to 28 weeks of age | Day 15 of the study  to 28 weeks of age | Day 15 of the study  to 28 weeks of age |
| Almeta^®^ ointment  every day until rash remission  , twice a day | Rinderon^®^-V ointment  every day until rash remission, twice a day | Rinderon^®^-V lotion  every day until rash remission  , twice a day |

Note:

When skin rash recurs, participants should re-apply TCSs on their body daily until they obtain remission of the skin rash.

If participants do not have remission from the skin rash by applying Rinderon^®^-V ointment for 7 days, they are permitted to use Fulumeta^®^ ointment.

When ointment is removed by washing or wiping, it can be re-applied.

Participants should frequently apply ointment to the area around the mouth to keep the skin around their lips covered with ointment.

5.6.4 Conventional treatment group: Standard treatment based on the Guidelines for the Management of Atopic Dermatitis (2016) (1) (Step-up reactive method)

Basic whole-body treatment

Participants will be followed as described below and administered basic whole-body treatment, except for scalp.

|  | Whole body except scalp |
| --- | --- |
| Registration day (Day 0) of the study  to 28 weeks of age | Hirudoid^®^ Soft ointment  Every day  twice a day |

Additional skin rash treatment

Participants are to apply TCSs as additional treatment as described below:

| Area | Face | | Body except scalp and face | | | Scalp |
| --- | --- | --- | --- | --- | --- | --- |
| Severity of skin rash | Less mild | Mild, moderate, and severe | Less mild | Mild and moderate | Severe | Any severity |
| Registration day (Day 0) of the study  to 28 weeks of age | Without additional treatment | Almeta^®^ ointment  every day until rash remission  once a day | Without additional treatment | Almeta^®^ ointment every day until rash remission  once a day | Rinderon^®^-V  ointment  every day until rash remission  once a day | Rinderon^®^-V valerate lotion until rash remission  once a day |

Note:

When skin rash recurs, participants should re-apply TCSs on their body daily until they obtain remission of the skin rash.

If participants do not have remission from the skin rash by applying Rinderon^®^-V for 7 days, they are permitted to use mometasone (Fulumeta^®^).

When ointment is removed by washing or wiping, it can be re-applied.

If study physicians judge participants do not obtain remission to AD 8 weeks after the entry, they can change the treatment to aggressive intervention treatment, which is treated as a deviation.

## 5.7 PERMITTED CONCOMITANT TREATMENT

Permitted concomitant treatments are described below. Investigators assess the concomitant treatment every study visit.

1. Inhaled, nasal, ocular, and ear drop steroids
2. Petrolatum (white petrolatum: Propeto^®^) ointment
3. Treatment for skin diseases except for AD

Zinc oxide ointment, dimethylisopropylazulene ointment (e.g., Azunol^®^ ointment 0.033%), antibiotic ointment (e.g., nadifloxacin ointment: Acuatim^®^ ointment 1%), antivirus ointment (e.g., acyclovir ointment: Zovirax^®^ ointment 5%), antifungal ointment (e.g., allylamine antifungal ointment: Lamisil^®^ cream 1%), keratolytic and dry skin ointment (e.g., urea ointment: Pastaron^®^ cream 10%), ointment-steroid combinations (e.g., killed bacteria-hydrocortisone combinations: Eksalb^®^; chloramphenicol fradiomycin sulfate ointment: Chlomy^®^-p ointment)

1. Laser therapy for pigmented nevi, angioma, and so forth.

## 5.8 NON-PERMITTED CONCOMITANT TREATMENT

Concomitant treatments described below are not permitted because of their effect on the efficacy of the study.

1. TCSs except study medications
2. NSAID topical ointment (e.g., Ibuprofen piconol: Staderm^®^ ointment)
3. Systemic (oral or intravenous) steroids
4. Immunosuppressive medications except topical/oral/intravenous steroids (cyclosporine, tacrolimus, and so forth)
5. Biologics except vaccinations (Infliximab, Etanercept, Tocilizumab, and so forth)
6. Immunoglobulin

## 5.9 STUDY COMPLETION AND DISCONTINUATION

5.9.1 STUDY COMPLETION

The study is complete when the oral food challenge tests at 28 weeks of age are performed. When primary outcome evaluation is finished, study treatment for a participant is completed and study physicians observe, conduct tests, and evaluate the participant at the time of the study completion.

5.9.2 STUDY DISCONTINUATION

Participants can discontinue the study if they meet any of the criteria described below:

Study physicians determine that the participants are unable to continue study treatment in PACI study due to exacerbation of AD

Participation in the study is discontinued because of adverse events

Proxies (parents or legal guardians) withdrawal from the study

Participants are unable to attend a study visit because of moving or hospital transfer

Death during study participation

PACI study is terminated

Study physicians determine that it is difficult to continue the study intervention for participants for reasons other than the above

# 6 STUDY ASSESSMENTS (Observation, test, and evaluation)

## 6.1 ASSESSMENT SCHEDULE

|  | Study treatment start | Study period | | | |
| --- | --- | --- | --- | --- | --- |
| Visit | 1 | 2 | 3 | 4 | 5 |
| Study duration | Entry  (Day 0) | 2 weeks after entry  (Day 14) | 4 weeks after entry  (Day 28) | 8 weeks after entry  (Day 56) | 28 weeks of age |
| Visit windows |  | ±6 days | ±7 days | ±7 days | ±14 days |
| Informed consent | X |  |  |  |  |
| Background information | X |  |  |  |  |
| Nutrition | X | X | X | X | X |
| Living environment | X | X | X | X | X |
| Body height | X | X | X | X | X |
| Body weight | X | X | X | X | X |
| Oral food challenge test  (hen’s egg) |  |  |  |  | X |
| IgE-mediated FA  by interview | X | X | X | X | X |
| Wheeze | X | X | X | X | X |
| Total IgE, egg white, ovomucoid, milk, wheat soy, peanut, and Ara h2-specific IgE and IgG4 antibodies in serum | X |  |  |  | X |
| EASI  by blinded physician | X | X | X | X | X |
| POEM  by caregivers |  | ✔ | ✔ |  | Every week |
| Disease-free days of AD |  | X | X | X | X |
| Amount of emollients and TCSs including rescue medication usage |  | X | X | X | X |
| Days of applying emollients and TCSs including rescue medication usage |  |  |  | Daily |  |
| IDQoL and DFI scores | X | X | X | X | X |
| Salivary cortisol |  | X | X | X | X |
| Concomitant therapy | X | X | X | X | X |
| Adverse events |  | X | X | X | X |
| Amount of emollients and TCSs used |  | X | X | X | X |

Measurement of specific IgG4 antibody titer is carried out at the Institute of Enzyme Research, University of Tokushima, and salivary cortisol is measured at the Center for Environmental Risk Research, National Institute for Environmental Studies (Supplement 12). The total IgE antibody titer and the specific IgE antibody titers are measured by the ImmunoCAP method provided by health insurance.

## 6.2 ASSESSMENT DETAILS

Study physicians investigate patient information from medical information as listed below. The PACI Study does not examine genetic features of participants.

(1) Study entry

Informed consent, birthday, sex, inclusion criteria, and exclusion criteria

Patient background: gestational age, body weight at birth, body height at birth, past medical history, race

Mother’s background: age at study entry, socioeconomic status (education), history of allergic diseases

Father’s background: socioeconomic status (education), history of allergic diseases

Siblings’ background: number of siblings, history of allergic diseases

Life style: pet ownership at home (dog, cat, and so forth), exposure to second-hand smoke

Nutrition: breastfeeding, milk formula, hypo-allergenic milk formula, solid food introduction, egg, cow’s milk, wheat, soy, and peanut intake, food restrictions during pregnancy and lactation

Body weight at study entry, body height at study entry

History of IgE-mediated FA by interview, history of wheeze by interview

Serum tests: total IgE antibodies titer in serum; egg white titer; ovomucoid, milk, soy, wheat, peanut and Ara h2-specific IgE antibody levels in serum; egg white titer; ovomucoid, milk, soy, wheat, peanut and Ara h2-specific IgG4 antibody levels in serum

Severity of AD: POEM, EASI by blinded physician

QoL: IDQoL and DFI

Concomitant medication/treatment

(2) Week 2, Week 4, Week 8

Life style: pet ownership at home (dog, cat, and so forth), exposure to second-hand smoke

Nutrition: breastfeeding, milk formula, hypo-allergenic milk formula, solid food introduction, egg, cow’s milk, wheat, soy, and peanut intake, food restrictions during pregnancy and lactation,

Body weight

History of IgE-mediated FA by interview, history of wheeze by interview

Severity of AD: POEM, EASI by blinded physician

AD disease-free days, Prescription volume/remaining amount/days used of emollients and TCS used including rescue medication used

Salivary cortisol, concomitant medication/treatment, adverse events

(3) 28 weeks of age

Life style: pet ownership at home (dog, cat, and so forth), exposure to second-hand smoke

Nutrition: breastfeeding, milk formula, hypo-allergenic milk formula, solid food introduction, egg, cow’s milk, wheat, soy, and peanut intake, food restrictions during lactation,

Body weight at study entry, body height at study entry

Food challenge test (hen’s egg): open method investigated by blinded physician at the age of 28 weeks. Pasteurized low egg powder 2.6 g (whole egg 10.4 g, egg protein 1.2 g) is given. Participants eat 0.1–0.5–2 g of the pasteurized egg powder every 40 minutes.

History of IgE-mediated FA by interview, history of wheeze by interview

Serum test: total IgE antibodies titer in serum; egg white titer; ovomucoid, milk, soy, wheat, peanut and Ara h2-specific IgE antibody levels in serum; egg white titer; ovomucoid, milk, soy, wheat, peanut and Ara h2-specific IgG4 antibody levels in serum

Severity of AD: POEM, EASI by blinded physician

AD disease-free days, Prescription volume/remaining amount/days used of emollients and TCS used including rescue medication used

Salivary cortisol, concomitant medication/treatment, adverse events

# 7 ADVERSE EVENTS

Adverse events are defined as any unfavorable symptoms and signs (including anomalies in laboratory test values) occurring in participants, with or without a causal relationship with interventional treatment. When an adverse event develops, study physicians promptly carry out necessary action (examination, treatment, discontinuation of study treatment, and so forth) and make an effort to ensure participant safety.

Study physicians check latest insert on the website of the Pharmaceuticals and Medical Devices Agency <http://www.info.pmda.go.jp/>. An adverse event to be evaluated is recorded in the medical record and electronic case report (eCRF).

## 7.1 COLLECTION OF ADVERSE EVENTS

In this study, we evaluate the following adverse events that occurred from the start of the study treatment to the completion or discontinuation of the study treatment. Adverse drug reactions are defined as any adverse events that cannot be denied relatedness to the study drugs (Hirudoid^®^ Soft ointment, Almeta^®^, Rinderon^®^-V, and Fulumeta^®^). Well-known adverse drug reactions are any events listed in the package inserts. Unknown adverse drug reactions are any events not listed on the package inserts.

(1) serious adverse events (SAEs) (It does not matter whether or not the events are related to the study treatment)

(2) well-known and unknown adverse drug reactions that occur in the study intervention would include those for Hirudoid^®^ Soft ointment, Almeta^®^, Rinderon^®^-V, and Fulmeta^®^

## 7.2 ASSESSMENT OF ADVERSE EVENTS

Investigators collect and assess adverse events by medical interview, physical examination, and skin diary every study visit. Study physicians assess whether or not participants have adverse drug reactions related to the study drugs. If SAEs and adverse drug reactions are discovered, study physicians record as listed below.

They record diagnosis or event, the day of occurrence, the severity (mild, moderate, or severe), the seriousness (serious or non-serious), the status of the study (continuation, extension, cessation, or stop), the procedure for adverse events (done or not, details if it occurs), the outcome of adverse events (resolved, improved, still present, improved with sequelae, death, unknown, reason if the evaluation is finished with unknown status), the day the physician assesses the status, and relatedness to the study (relatedness denied or relatedness that cannot be denied, evidence of judgment if it is relatedness that cannot be denied).

7.2.1 SEVERITY OF ADVERSE EVENTS

If adverse events are observed, study physicians assess the severity for the most serious condition during the occurrence of the adverse event at three levels, as described below.

1. Mild: signs or symptoms observed that do not need to be treated nor impact on daily activities
2. Moderate: clinical signs or symptoms that need to be treated or impact on daily activities
3. Severe: clinically serious signs or symptoms and events that cause participants to be unable to perform daily activities

7.2.2 OUTCOME OF ADVERSE EVENTS

The outcome of adverse events is assessed at six levels (resolved, improved, still present, improved with sequelae, death, and unknown). If event resolution, improvement, or death is observed, the day of outcome assessment is the day when the event is observed. If the outcome is determined as still present or unknown and participant follow-up to be terminated[[please confirm this meaning]], the day of the outcome assessment is the day when the physicians assess the outcome. Investigators must record the reasons why they remove (terminate) a participant from the study following adverse events or why the adverse events are unknown on the medical chart and eCRF.

7.2.3 SERIOUS ADVERSE EVENTS

Serious adverse events (SAEs) are defined as follows:

1. Death
2. Disability
3. Life-threatening event
4. Disability/incapacity-threatening event
5. Hospitalization, or prolonged existing hospitalization
6. Other serious conditions
7. Congenital or acquired anomaly

For (5), a hospital admission (planned surgery, examination, and so forth) that is planned before the study entry is not included as a hospitalization. However, a new occurrence of an adverse event during the hospitalization is included.

7.2.4 RELATEDNESS TO STUDY INTERVENTIONS

For SAEs, the relatedness to the study intervention is assessed in two categories. If the adverse event is determined to be non-related to the study intervention, the reason is recorded on the medical chart and eCRF.

1. Relatedness can be denied: In cases where it is impossible for the temporal relatedness between adverse event development and study treatment or when the cause of adverse event development can be reasonably explained medically except for study treatment
2. Relatedness cannot be denied: adverse events that are not applicable as above

## 7.3 REPORTING OF ADVERSE EVENTS

Both SAEs that are considered to be related to the study intervention and SAEs that are considered to be unrelated to the study intervention will be reported to the Pharmaceuticals and Medical Devices Safety Information Reporting System [Article 68 of the Pharmaceutical Affairs Law as applied under Article 10(2)]. Principal study investigator reports to the Minister of Health, Labor and Welfare if a serious adverse event is unpredictable and the direct relatedness to the study cannot be denied. In addition, study physicians report spontaneously to the manufacturer in accordance with the provisions of each research implementation facility for the purpose of cooperating with the manufacturer 's voluntary report [Article 68 of the Pharmaceutical Affairs Law as applied under Article 10(1)]. Investigators will input the SAE and adverse drug reaction information into the medical chart and the eCRF.

7.3.1 IMMEDIATE REPORTING OF ADVERSE EVENTS

SAEs (7.2.3) will be reported immediately. If an SAE is observed, study physicians will report the SAE to the head of each study investigational site. The head of the study investigational site must fill out and submit the immediate adverse event report (supplement 3) (or another official document of each investigational site) to the head of each institute and study-coordinating center by email within 72 hours. Study physicians must fill out and email the serious adverse event report (supplement 4) to the study-coordinating center and the data center within 7 days. The study-coordinating center will report the serious adverse event report to the principle investigator and data center.

7.3.2 ROUTINE REPORTING OF ADVERSE EVENTS

If adverse events that are subject to routine reporting are observed, study physicians will input the event information into the eCRF. All events are followed until the event outcome becomes clear and is input into the CRF. If the event cannot be followed for some reason, the study physicians will record the reason on the medical chart and the eCRF.

7.3.3 REPORTING TO THE INDEPENDENT DATA MONITORING COMMITTEE

The study-coordinating center will email the SAE reported from investigational sites immediately to the independent data monitoring committee (IDMC) as soon as the study-coordinating center recognizes the SAEs, and they will ask the IDMC to investigate whether consideration and action in response to the SAE is appropriate. The IDMC investigates the reports and makes a recommendation about the SAEs to the principal investigator. The principal investigator makes a decision regarding study continuation and informs the investigational sites immediately of the decision. If the principal investigator temporarily stops recruitment into the study, he/she will immediately inform the investigational sites of this information and take action.

# 8 ENDPOINTS

## 8.1 PRIMARY ENDPOINT

1. Oral food challenge-proven IgE-mediated hen’s egg allergy at the age of 28 weeks

Justification for the primary endpoint

This study evaluates the presence of IgE-mediated hen’s egg allergy as primary endpoint because hen’s egg is the most common causal FA in Japan.

The timing of oral food challenge tests for hen’s egg is set at the age of 28 weeks because we decided to make a recommendation for participants in our study to ingest hen’s egg starting at 6 months of age based on the past study results reported by Natsume *et al.*

A double-blind, placebo-controlled oral food challenge is the gold standard for diagnosis of IgE-mediated FA to reduce complaints by patients and psychogenic reactions. Because the participants in our study are infants who are at the age of 28 weeks, they are very unlikely to show subjective complains and it is rare that psychological reaction has an effect on the evaluation for OFC. An open method of a food challenge test is common among infants under 1 year old. An open method of a food challenge test can be performed easily, and it results in less of a burden for infants and their caregivers and is expected to be an adequate reliable assessment. Therefore, we decided to use an open method for oral food challenge tests in the study.

## 8.2 SECONDARY ENDPOINTS

EFFICACY ENDPOINTS

1. Food challenge test scores at the age of 28 weeks
2. Total IgE antibody titer in serum at the age of 28 weeks
3. Egg white, ovomucoid, milk, soy, wheat, and peanut-specific IgE antibody titers in serum at the age of 28 weeks
4. Egg white, ovomucoid, milk, soy, wheat, and peanut-specific IgG4 antibody titers in serum at the age of 28 weeks
5. Eczema Area and Severity Index (EASI) scores at 2, 4, and 8 weeks after study entry and at the age of 28 weeks
6. Patient Oriented Eczema Measure (POEM) scores weekly through the study
7. Percentage of disease-free days throughout the study
8. Dose of rescue medication used throughout the duration of the study
9. Infants’ Dermatitis Quality of Life Questionnaire (IDQoL) at 2, 4, and 8 weeks after study entry and at 28 weeks of age
10. Family Impact of Childhood Eczema Questionnaire (DFI) at 2, 4, and 8 weeks after study entry and at 28 weeks of age
11. Cumulative incidence of IgE-mediated FA assessed by a doctor’s interview during the study
12. Cumulative incidence of wheezing assessed by a doctor’s interview during the study

Justification for secondary endpoints (efficacy)

(1) Scoring of the food challenge test has already been recommended in the American Academy of Allergy, Asthma & Immunology – European Academy of Allergy and Clinical Immunology PRACTALL consensus report (65).

(2) (3) To evaluate allergy sensitization

(4) To evaluate immune tolerance against hen’s egg

(5) (6) To evaluate the activity and severity of AD

(7) (8) To evaluate the disease control

(9) (10) To evaluate the QoL

(11) To evaluate the prevalence of other IgE-mediated FA except hen’s egg

(12) To evaluate the prevalence of signs related to asthma

SECONDARY ENDPOINTS (SAFETY)

1. Serious adverse events
2. Adverse drug reactions
3. Salivary cortisol concentrations at 2, 4, and 8 weeks after study entry and at the age of 28 weeks
4. Body weight and body height at the age of 28 weeks

Justification for secondary endpoints (safety)

(1) (2) To evaluate safety of the study drugs

(3) To evaluate the effect on adrenal function by adverse drug reactions to TCSs

(4) To evaluate the effect of adverse drug reactions to TCSs on growth

ADHERENCE EVALUATION

(1) TCS adherence index in accordance with the protocol

(2) Dose of TCS used during the study

Justification for adherence evaluations

(1)(2) To evaluate adherence to the protocol

# 9 STATISTICS

## 9.1 SAMPLE SIZE

For sample size calculation, the proportion of egg allergy in early aggressive intervention and conventional treatment groups are estimated to be 20% and 30%, respectively. With a one-sided significance level of 0.025, 581 participants are needed to provide 80% statistical power. One interim analysis examining efficacy and safety is planned when almost half the participants are finished their trial assessment. Considering the interim analysis, 614 participants are needed. We expect a drop of about 5%, and set the target number of study participants to 650.

## 9.2 ANALYSIS SET

The following data set is used for analysis. The target analysis of the primary endpoint of efficacy is defined as Full Analysis Set (FAS).

1. Full Analysis Set (FAS)

The group excluding the participants as follows from all the participants who are randomized

- - Participants who have never received intervention
  - Participants who have not yielded data after the start of the study treatment
  - Participants who are found to have violated eligibility criteria after received intervention t

1. Per Protocol Set (PPS)

Of the FAS, the group consisting of participants without significant deviation from the implementation plan

1. Target group for safety analysis

The group consisting of all participants who have undergone the study treatment at least once

## 9.3 STATISTICAL ANALYSIS

9.3.1 SUMMARY OF THE BACKGROUND INFORMATION

The categorical variables (gender, medical history, past medical history, and so forth) are summarized using the frequency and proportion. The continuous variables (age, height, weight, and so forth) are summarized using the average, standard deviation, median, quartile range, minimum, and maximum. All variables are shown in each group and stratification: the number of weeks after birth.

9.3.2 PRIMARY ENDPOINT ANALYSIS

Statistical analysis will be performed following the intention to treat (ITT) principle, where participants will be analyzed as they were randomized, not as they were treated. The primary analysis is performed using FAS, and secondarily using PPS. The participants who have missing data for the presence of IgE-mediated hen’s egg allergy are defined as having IgE-mediated hen’s egg allergy and included in the FAS analysis. The participants who have missing data for the presence of IgE-mediated hen’s egg allergy are excluded from the PPS analysis. To verify the study hypothesis, “the aggressive intervention group has lower prevalence of IgE-mediated hen’s egg allergy compared to the conventional treatment group”, the main comparison is the null hypothesis that the percentage of IgE-mediated hen’s egg allergy is higher in the aggressive treatment group than in the conventional group" and the alternative hypothesis is that the percentage of IgE-mediated hen’s egg allergy is lower in the aggressive treatment group than in the conventional group. The *p* value is calculated by the difference in percentage with a one-sided test. The significance level is 0.025. The difference and ratio of these proportions and their 95% confidence intervals for each intervention group will also be calculated.

9.3.3 SECONDARY ENDPOINTS ANALYSIS

All secondary endpoints will be analyzed using FAS and secondarily using PPS. The details of statistical methods are described in the statistical analysis plan.

9.3.4 INTERIM ANALYSIS

Interim analysis is conducted with the aim of preventing disadvantages to participants due to excessive efficacy and safety procedures unintended in study treatment. The interim analysis is performed when almost half of the participants finish their assessments for the primary endpoint. The interim analysis is done for primary endpoint and safety endpoints. The study registration for study participants does not stop while conducting the interim analysis. The analysis of primary endpoint is performed along with 9.3.2 primary endpoints analysis. The stopping boundary for the analysis of primary endpoint will be calculated based on the O’Brien–Fleming type with a Lan–DeMets α and βspending function at the actual information time. For example, the significance levels of effective discontinuation and invalid discontinuation are 0.0015 and 0.2883, respectively, with the interim analysis for 307 participants who are half of the planned study participants. The interim analysis is conducted by a trial statistician of the interim analysis under a closed situation. The statistician prepares a report of interim analysis results and submits it to the independent data monitoring committee. The results of the interim analysis will be evaluated by the members of the independent data monitoring committee to investigate whether or not the study continues, and the investigation result report is submitted to the principle investigator.

# 10 DATA MANAGEMENT

Data management is carried out according to the data management plan determined separately.

# 11 ETHICAL CONSIDERATIONS

This study follows the Declaration of Helsinki Ethical Principles for Medical Research Involving Human Subjects and the Ethical Guidelines for Medical and Health Research Involving Human Subjects (2014 December 22, the Japanese Ministry of Education, Ministry of Health, Labour and Welfare Notification No. 3).

## 11.1 ETHICAL REVIEW

This study is performed with the permission of the dean of each institute after it has been reviewed and approved by each Institutional Review Board (IRB).

## 11.2 INFORMED CONSENT

Prior to the start of the study, the investigators will obtain voluntary written consent from the proxy person (parents or legal guardians) after adequately explaining the explanatory items listed below based on the explanatory document (supplement 5) with obtaining sufficient understanding for the study. The investigators will inform the proxy person (parents or legal guardians) that all participants are free to withdraw at any time from study participation and that the proxy person’s (parents or legal guardians) refusal or withdrawal from participation in the study does not cause any disadvantage to participants. Investigators and the proxy persons’ (parents or legal guardians) sign their names and the date on the consent form when investigators obtain the informed consent. Withdrawing consent never suffers a disadvantage.

Explanatory items

- The title of the study
- The name of the study investigational site and the head of the study
- Purpose and necessity of the study
- Study methods
- Expected participants
- Expected benefits and disadvantages
- Voluntary participation and the right to withdraw
- Share study protocol
- Privacy and confidentiality
- Sample and data management (storage and discard)
- Study funding and conflict of interest
- Communication with participants
- Cost burden and reward
- Matters concerning the case of unapproved (medicine/device) study
- Handling of genetic characteristics and so forth
- Compensation for health damage
- How to deal with samples and data for future study
- Monitoring and audit

## 11.3 WITHDRAWAL OF CONSENT

If the proxy persons (parents or legal guardians) request to withdraw their consent for study participation, investigators will follow the procedure described below (supplement 6).

1. Investigators will collect the consent withdrawal form (supplement 6) from the proxy person (parents or legal guardians) and review the form.
2. Investigators will instruct the study-coordinating center to let the participants withdraw from the study and to discard the samples.
3. The study-coordinating center will instruct the sample storage center to discard the samples.
4. The storage center will inform when the samples have been discarded by completing the form (supplement 7).
5. The study-coordinating center will inform the head investigator of the investigational site by phone or email when the samples have been discarded.

## 11.4 PRIVACY AND CONFIDENTIALTY

Participants’ privacy and confidentiality will be respected throughout the study. The heads of the investigational sites must establish a system of safety management for privacy and confidentiality.

For publication, confidentiality of participants’ identifying information is required. All data can only be used for the study purposes. If the data is supposed to be used for another purpose outside of this study, another informed consent form is required from the proxy person (parents or legal guardians).

Personal information of participants is anonymous (modified and controlled samples and information for any participants which are not be judged by others). The personal information is appropriately managed by the personal information manager in each study period using the personal information correspondence table and the participant identification number, and not shared with the study-coordinating center, the data center, sample storage center, laboratory test centers, and trial statisticians. The study physicians give the participant's unique anonymization number to the participant and manage the sample specimen with the anonymization number without name, address, telephone number, and so forth. For identification of participants among the study-coordinating center, sample storage center, and laboratory test centers, the anonymization number is used. The sample storage center and the laboratory test centers strictly store and manage the received sample specimen. Study members do not input personal information that can identify patients in the Electronic Case Report (eCRF). Participant identification number is used for identification of individuals among the study-coordinating center, sample storage center, and laboratory test centers. Study physicians use the participant identification number check sheet (supplement 8), recording patient ID at the study site, the participants’ study number, anonymous number, sex, and day of study registration. The participant identification number check sheet is strictly stored within a locked room, formed as the original record by handwriting. In handling of the electronic case report (eCRF), confidentiality of privacy must be considered.

## 11.5 EXPECTED BENEFITS AND DISADVANTAGES

This study is a randomized controlled trial to assess the efficacy of early aggressive intervention (proactive method) with TCSs for infantile AD, compared with conventional treatment for infantile AD based on the Guidelines for the Management of Atopic Dermatitis (2016) (1) (reactive method). There is not enough evidence to show that either one is superior, and the implementation of randomization is under prerequisite that the equipoise of efficacy and safety of both groups exists. Because both interventions are performed daily, there are neither specific benefits nor disadvantages for participants. During the study period, participants can not use TCSs other than trial drugs because they are prohibited as concomitant treatment. Although participants are required to apply to the skin only TCSs that are trial drugs, participants can apply medium, strong, or very strong TCSs, the cost of which is covered for all infants with AD and is not disadvantageous. To reduce the burden of the participants and the proxies (parents or legal guardians), there is saliva sampling without invasiveness. Although the frequency of blood tests is usually within the scope of medical examination, an extra 2.5 mL is collected for laboratory tests and serum preservation. In addition, the proxies (parents or legal guardians) have a duty to write diaries and questionnaires and to bring the rest of the trial drugs. If adverse events related to the trial drugs occur, or study physicians determine that participants need to discontinue their participation, study physicians stop the study immediately, change to different medications, and the participants must obtain a different treatment plan and clinical examinations. The study physicians must ensure the safety and best practice for participants.

# 12 PROTOCOL VIOLATIONS AND DEVIATIONS

A protocol deviation is non-compliance with the approved study protocol related to study treatment, clinical laboratory tests, and so forth. The data center will report any possible deviations that are beyond the applicable deviations defined in the protocol before study initiation to the study-coordinating center. These deviations are categorized as listed below via the steering committee investigation.

## 12.1 VIOLATIONS

A protocol violation is defined as a deviation from the protocol that meets several criteria listed below and is judged as a clinically inappropriate event by investigational sites or investigators listed as follows:

1. impacts on the primary endpoint assessment
2. an intentional or systematic event
3. risky or significantly deviated event.

All violations will be described in the final report in principle when publishing the paper.

## 12.2 DEVIATIONS

12.2.1 UNEXPECTED DEVIATION

Unexpected deviations are not protocol violations (12.1) nor acceptable deviations (12.2.2). If the study physician judges that it is not possible to maintain remission of atopic dermatitis in the standard treatment group, it will be possible to start the same protocol as the early aggressive treatment group later than 8 weeks after enrollment, and such a case is defined as a deviation.

Deviations are described in central monitoring reports. If there are many specific deviations, the results of the deviations will be mentioned in the final report.

12.2.2 ACCEPTABLE DEVIATION

Acceptable deviations are decided by the steering committee, the principle investigator and/or the study-coordinating center under discussion with the data center and defined before and/or after study initiation. Acceptable deviations will not be mentioned in monitoring reports.

# 13 QUALITY CONTROL AND QUALITY ASSURANCE

## 13.1 MONITORING

This study follows the Standard Operating Procedure for monitoring and audit of clinical studies in the National Center for Child Health and Development and for conduct of monitoring to determine that the study is performed safely and in accordance with the protocol. The data from the eCRF are checked by central monitoring. The data center reports regularly on data monitoring results and they are submitted to the principle investigator. The principal investigator submits the report to the Steering Committee, the Independent Data Monitoring Committee, and the head of each facility. As a result of central monitoring, if the principle investigator deems it necessary, on-site monitoring will be carried out. The monitoring system and the implementation plan are described in Supplements 9 and 10.

## 13.2 AUDIT

This study will not be audited.

## 13.3 STORAGE AND DISCARD OF DATA AND SAMPLES

All records about participant's consent, the participant identification number check sheet, data to be entered in the electronic case report (eCRF) (examination data, treatment diary, and so forth), authorization form of the IRB, and recorded documents by the study investigational site are kept by the head of each study investigational site. For the management at the Center for Development and Medical Care, they are kept in a keyed cabinet at the Division of Allergy (Research Building 4th Floor). The storage duration will be 5 years after the study completes or stops. Laboratory test centers will discard all samples as soon as they are tested. The sample storage center will store all serum samples until the samples are used completely, following another study plan. The stored sample at the sample storage center is used according to a separately determined research plan and stored until the end of the retention period of the participant identification number check sheet or until the sample is used up, which ever is earliest.

## 13.4 HANDLING IN CASES WHERE SAMPLES AND INFORMATION IS LIKELY TO BE USED FOR FUTURE MEDICAL PRACTICE

Samples and information may be used for future study not identified at the present time, such as future follow-up survey or use for other study. In that case, the study plan will again be fixed and approved by the IRB and samples and information will be used.

## 13.5 DIRECT OBSERVATION FOR THE ORIGINAL DOCUMENTS

Investigators and investigational sites permit members of the monitoring group and the IRB to examine all relevant documents directly and study members corporate for direct observation.

# 14 CONFLICT OF INTEREST AND FUNDING ORGANIZATION

## 14.1 CONFLICT OF INTEREST

This study is performed based on the decision by the principal investigator and steering committee regarding the study plan, implementation, analysis, and publication. The investigators follow the management policy for conflict of interest by each institute and declare any conflicts of interest.

## 14.2 RELATIONSHIP WITH FUNDING ORGANIZATION

This study is funded by the Practical Research Project for Allergic Diseases and Immunology (Research on Allergic Diseases and Immunology), Japan Agency for Medical Research and Development (AMED). The funder for the study is not involved in planning, implementing, analyzing, or publishing the study.

# 15 COST BURDEN AND COMPENSATION FOR DAMAGE TO PARTICIPANTS’ HEALTH

## 15.1 COST BURDEN

Laboratory tests and prescriptions for study drugs, which are used within daily clinical practice, are covered by medical insurance. The salivary cortisol test and specific IgG4 antibody test by the DCP method that are not covered by medical insurance and specimen transportation expenses are contributed from the research funding sources. Therefore, the participants and their parents will not have to pay for additional medical cost. The participants and their parents will obtain a Quo card (1,000 yen) at 2, 4, and 8 weeks from entry and a Quo card (2,000 yen) at 28 weeks of age.

## 15.2 COMPENSATION FOR DAMAGE TO PARTICIPANTS’ HEALTH

If health damage occurs, treatments must be performed in agreement with the participant’s medical insurance as well as with daily medical practice. For this study, all of the personnel involved are covered by research insurance in case compensation of health damage caused to the participants is required. If health damage (disability) to the participants results from the study, this research insurance will pay the medical care cost for the damage and bear the legal liability.

# 16 PUBLICATION POLICY AND ATTRIBUTION OF PRODUCTIONS

# 16.1 CLINICAL TRIALS REGISTRY

The study will be registered on UMIN-CTR (http://www.umin.ac.jp/ctr/index-j.htm). The registry is completed before the first participant is recruited for the study. The study-coordinating center will manage the registry.

## 16.2 PUBLICATION POLICY AND ATTRIBUTION OF PRODUCTIONS

Presentations and publications about the study should follow the instructions as described below.

The final report will be submitted to an international journal after the final analysis is complete. Results of a study analysis that are not planned in the protocol can be presented if the principal investigator and the steering committee approve the presentation. The principal investigator and the members of the study-coordinating center are able to give presentations to promote the study, and to review the study information that does not include results of the final analysis if the principal investigator approves the presentation. As a general rule, the first author of the publication of the main research results shall be the head of the study-coordinating center, followed by the principal investigator, one statistician, one data center personnel, and members of the steering committee. Below that, in accordance with the instruction of the journal, the head of the investigational site who will register 50 participants or more becomes a co-author and co-author sequence will be made in order of the number of registered participants. Other co-authors will be properly decided by the principle investigator according to the author's requirements of the International Committee of Medical Journal Editors. If it exceeds the number of co-authors over the journal requirements because the PACI Study is a multicenter trial, the co-authors belong to the PACI Group members and are qualified as a co-author. All authors are the only people who will review the manuscript based on the final report before its submission. If there is no agreement on discussion of the final manuscript, the principle investigator is allowed not to include that researcher as a co-author after the approval by the steering committee. The study investigators who do not meet the author’s eligibility criteria are listed in the acknowledgement section. Because there is a possibility that the conference presentation may be repeated several times, it is possible to give a presentation from the head of the study-coordinating center, principle investigator, and head of the study investigational group with many participants. The presenter is decided by the principle investigator with approval of the steering committee chairperson. However, for presenting in the conference, the principle investigator is responsible for preparations for presentation and contents of presentation, and the study-coordinating center contacts the data center in principle. Presenters other than the members of the study-coordinating center can not receive the data and the statistical results of the study directly from the data center without approval by the principal investigator.

All productions from the PACI study belong to the PACI study group. The details will be decided through discussion by the steering committee.

## 16.3 ACCESS TO DATA

The data collected belongs to the principal investigator of the PACI Study. The principal investigator, study-coordinating center, steering committee, data center, and study statistician are permitted to access to the data in this study. If the journal we submit to requests study data sharing, we will follow the study instructions.

# 17 STUDY COMPLIANCE AND AMENDMENT

All study personnel will ensure human rights are maintained in accordance with the study protocol. In case of amendment and/or revision for the study protocol, explanatory document, and consent document, IRB approval is required. If any investigational site needs to change a particular study plan, the head investigator at the investigational site can revise the study protocol of the investigational site with the agreement of the principle investigator.

17.1 STUDY COMPLETION

The head investigator at each investigational site will submit the final report to the dean, IRB of the investigational sites, and the principal investigator of the study as soon as the study is complete.

17.2 STUDY STOP AND DISCONTINUATION

1. The IDMC will discuss the validity of continuing the study if needed. If the IDMC decides that study continuation is not appropriate, the IDMC will discuss stopping or discontinuing the study with the principal investigator. If the principal investigator decides the study must stop following discussion with the IDMC, the principal investigator will instruct the investigators on how to take action for the participants of the study. Each head investigator at the investigational site will report the details to the dean and the IRB of the investigational site, follow the decision by the IRB, and take action for the participants in the study.
2. If the IRB of the investigational site instructs the study to stop, the investigator of the investigational site will report the details to the principal investigator. The principal investigator will discuss the validity of the study continuation. If the investigators at the investigational site stop the study after instructions from the IRB, the investigators will report the details to the dean of the investigational site in a document.
3. The principal investigator will discuss the validity of continuing the study if the criteria are:
4. Any serious events and/or information about quality, safety, and efficacy of study drugs
5. It is difficult to recruit expected participants, in his/her judgment
6. The purpose of the study will be achieved before completing participant recruitment and study duration
7. Although the IDMC or the IRB decides whether the study protocol must be changed, the principal investigator judges whether it is unacceptable.

17.3 DEVIATION FROM THE STUDY PROTOCOL

Investigators are able to make a deviation from or amendment to the study protocol before the principal investigator and the IRB approve if there is a compelling reason, such as avoiding emergent accidents. If a protocol revision is needed, the investigators must submit the revised plan and obtain approval from the principal investigator, the dean of the institutional site, and the IRB. All deviations and their explanations will be recorded.

17.4 CHANGES TO THE STUDY PROTOCOL

17.4.1 CATEGORIES OF THE CHANGES TO THE STUDY PROTOCOL

Study changes have two categories: amendment and revision, as described below.

1. Amendment: there is increasing risk to the study participants and effects on the primary endpoint. The protocol amendment must be approved by the steering committee and the IRB. The day of the approval by the IRB is entered onto the protocol cover page.
2. Revision: there is no increasing risk to the study participants and no effects on the primary endpoint. The protocol revision must be approved by the IRB. The revision will be reported to the principal investigator and the steering committee, although the revision will be judged by the steering committee. Each investigational site follows the rules of the investigational site for an approval by the IRB. The day of the approval is entered onto the protocol cover page.

17.4.2 AMENDEMENT AND REVISION OF THE STUDY PLAN APPROVAL BY THE IRB

Amendment to the protocol must be approved by the IRB, if amendment of study plan and/or explanatory document is approved by the steering committee during the conduction of the study. In case of revision, not amendment, the requirement of the approval by the IRB follows the rules of each investigational site. If the protocol amendment is approved by the IRB of the investigational sites, each head investigator of the institutional sites will send the copy of the IRB approval document to the study-coordinating center. The head investigators will keep the document and the study-coordinating center will also keep a copy of the document.

# 18 STUDY ORGANIZATION

Principal Investigator:

Yukihiro Ohya

Chief Physician - Division of Allergy, Department of Medical Specialties

National Center for Child Health and Development

2-10-1 Okura, Setagaya-ku, Tokyo 157-8535, Japan

E-mail: ohya-y@ncchd.go.jp

Tel.: +81-3-3416-0181 (ext: 7021); Fax: +81-3-3415-9260

Study-Coordinating Center:

Kiwako Yamamoto-Hanada

Attending Physician - Division of Allergy, Department of Medical Specialties

National Center for Child Health and Development

2-10-1 Okura, Setagaya-ku, Tokyo 157-8535, Japan

E-mail: allergy_research@ncchd.go.jp

Tel.: +81-3-3416-0611; Fax: +81-3-3415-9260

Steering Committee

Chairperson:

Hirohisa Saito

Department of Allergy and Immunology, National Research Institute for Child Health and Development, Tokyo, Japan

E-mail：saito-hr@ncchd.go.jp

Committee members:

Yukihiro Ohya

Chief Physician, Division of Allergy, Department of Medical Specialties, National Center for Child Health and Development, Tokyo, Japan.

E-mail: ohya-y@ncchd.go.jp

Tohru Kobayashi

Director, Division of Clinical Research Planning, Department of Development Strategy, Center for Clinical Research and Development, National Center for Child Health and Development, Tokyo, Japan

E-mail：kobayashi-tr@ncchd.go.jp

Osamu Natsume

Assistant Professor, Department of Pediatrics, School of Medicine, Hamamatsu University, Shizuoka, Japan.

E-mail：natsumeo@hama-med.ac.jp

Kumiko Morita

Assistant Professor, Department of Pediatrics, School of Medicine, Keio University, Tokyo, Japan

E-mail：morita-k@keio.jp

Kiwako Yamamoto-Hanada

Attending Physician, Division of Allergy, Department of Medical Specialties, National Center for Child Health and Development, Tokyo, Japan.

E-mail：yamamoto-k@ncchd.go.jp

Mayako Saito

Physician, Division of Allergy, Department of Medical Specialties, National Center for Child Health and Development, Tokyo, Japan.

E-mail：saito-myk@ncchd.go.jp

Independent Data Monitoring Committee

Chairperson:

Shuichi Ito

Professor, Department of Pediatrics, Graduate School of Medicine Yokohama City University

E-mail：itoshu＠yokohama-cu.ac.jp

Committee members:

Rin Nishi

Physician, Yutenji Family Clinic Ten, Tokyo, Japan

E-mail：rin-rin-rinco@hotmail.co.jp

Ayano Takeuchi

Lecturer, Department of Preventive Medicine and Public Health, School of Medicine, Keio University

E-mail：ayanotakeuchi@keio.jp

Data Center・Central Monitoring

Naoko Shimomura

Data manager, Division of Data Management for Clinical Research, department of Data Management, Center for Clinical Research and Development, National Center for Child Health and Development, Tokyo, Japan

E-mail：dcc@ncchd.go.jp

Miwako Seike

Data manager, Division of Data Management for Clinical Research, department of Data Management, Center for Clinical Research and Development, National Center for Child Health and Development, Tokyo, Japan

E-mail：dcc@ncchd.go.jp

Individual Information Manager

Kayo Kakimura

Director-General of the Budget Bureau, Strategic Planning Office, National Center for Child Health and Development, Tokyo, Japan

E-mail：takimura-k@ncchd.go.jp

Trial Statistician

Masashi Mikami

Division of Biostatistics, Department of Data Management, Center for Clinical Research and Development, National Center for Child Health and Development, Tokyo, Japan

E-mail：mikami-ms@ncchd.go.jp

Laboratory test centers

Hiroshi Kido

Professor, Division of Enzyme Chemistry, Institute of Enzyme Research, Tokushima University, Tokushima, Japan

E-mail： kido@tokushima-u.ac.jp

Shoji Nakayama

Director, Centre for Health and Environmental Risk Research, National Institute for Environmental Studies, Ibaraki, Japan.

E-mail：fabre@nies.go.jp

Sample storage center

Aki Washizuka

Division of Allergy, Department of Medical Specialties, National Center for Child Health and Development, Tokyo, Japan.

E-mail：washizuka-a@ncchd.go.jp

External Evaluation Committee

Hywel Williams

Professor of Dermato-Epidemiology and Co-Director of the Centre of Evidence Based Dermatology, University of Nottingham, UK

Email: Hywel.williams@nottingham.ac.uk

Investigational sites

National Center for Child Health and Development

Hamamatsu University Hospital

Nagoya Medical Center

Sagamihara National Hospital

National Mie Hospital

University Hospital Kyoto Prefectural University of Medicine

Fujita Health University Hospital

Osaka Habikino Medical Center

Yamaguchi University Hospital

Keio University Hospital

Banbuntane Hotokukai Hospital

Kindai University Hospital

Chiba University Hosipital

Tohoku Medical and Pharmaceutical University Hospital,

Showa General Hospital, and Saitama City Hospital.

# 19 REFERENCES

1. Saeki H, Nakahara T, Tanaka A, Kabashima K, Sugaya M, Murota H, et al. Clinical Practice Guidelines for the Management of Atopic Dermatitis 2016. The Journal of dermatology. 2016:n/a-n/a.

2. Irvine AD, McLean WH, Leung DY. Filaggrin mutations associated with skin and allergic diseases. The New England journal of medicine. 2011;365(14):1315-27.

3. Palmer CN, Irvine AD, Terron-Kwiatkowski A, Zhao Y, Liao H, Lee SP, et al. Common loss-of-function variants of the epidermal barrier protein filaggrin are a major predisposing factor for atopic dermatitis. Nat Genet. 2006;38(4):441-6.

4. Howell MD, Kim BE, Gao P, Grant AV, Boguniewicz M, Debenedetto A, et al. Cytokine modulation of atopic dermatitis filaggrin skin expression. The Journal of allergy and clinical immunology. 2007;120(1):150-5.

5. Horimukai K, Morita K, Narita M, Kondo M, Kitazawa H, Nozaki M, et al. Application of moisturizer to neonates prevents development of atopic dermatitis. The Journal of allergy and clinical immunology. 2014;134(4):824-30 e6.

6. Simpson EL, Chalmers JR, Hanifin JM, Thomas KS, Cork MJ, McLean WH, et al. Emollient enhancement of the skin barrier from birth offers effective atopic dermatitis prevention. The Journal of allergy and clinical immunology. 2014;134(4):818-23.

7. Kubo A, Nagao K, Yokouchi M, Sasaki H, Amagai M. External antigen uptake by Langerhans cells with reorganization of epidermal tight junction barriers. J Exp Med. 2009;206(13):2937-46.

8. Yoshida K, Kubo A, Fujita H, Yokouchi M, Ishii K, Kawasaki H, et al. Distinct behavior of human Langerhans cells and inflammatory dendritic epidermal cells at tight junctions in patients with atopic dermatitis. The Journal of allergy and clinical immunology. 2014;134(4):856-64.

9. Deckers IAG, McLean S, Linssen S, Mommers M, van Schayck CP, Sheikh A. Investigating International Time Trends in the Incidence and Prevalence of Atopic Eczema 1990–2010: A Systematic Review of Epidemiological Studies. PloS one. 2012;7(7):e39803.

10. Ito J, Fujiwara T. Breastfeeding and risk of atopic dermatitis up to the age 42 months: a birth cohort study in Japan. Ann Epidemiol. 2014;24(4):267-72.

11. Rudikoff D, Lebwohl M. Atopic dermatitis. Lancet. 1998;351(9117):1715-21.

12. Bohme M, Wickman M, Lennart Nordvall S, Svartengren M, Wahlgren CF. Family history and risk of atopic dermatitis in children up to 4 years. Clinical and experimental allergy : journal of the British Society for Allergy and Clinical Immunology. 2003;33(9):1226-31.

13. Thomsen SF. Epidemiology and natural history of atopic diseases. Eur Clin Respir J. 2015;2.

14. Shoda T, Futamura M, Yang L, Yamamoto-Hanada K, Narita M, Saito H, et al. Timing of eczema onset and risk of food allergy at 3 years of age: A hospital-based prospective birth cohort study. J Dermatol Sci. 2016.

15. Eichenfield LF, Boguniewicz M, Simpson EL, Russell JJ, Block JK, Feldman SR, et al. Translating Atopic Dermatitis Management Guidelines Into Practice for Primary Care Providers. Pediatrics. 2015;136(3):554-65.

16. Potency of Topical Corticosteroids (UK Classification). 2015.

17. Long CC, Mills CM, Finlay AY. A practical guide to topical therapy in children. The British journal of dermatology. 1998;138(2):293-6.

18. Wollenberg A, Ehmann LM. Long term treatment concepts and proactive therapy for atopic eczema. Ann Dermatol. 2012;24(3):253-60.

19. Bieber T. Atopic dermatitis. Ann Dermatol. 2010;22(2):125-37.

20. Hanifin J, Gupta AK, Rajagopalan R. Intermittent dosing of fluticasone propionate cream for reducing the risk of relapse in atopic dermatitis patients. The British journal of dermatology. 2002;147(3):528-37.

21. Fukuie T, Hirakawa S, Narita M, Nomura I, Matsumoto K, Tokura Y, et al. Potential preventive effects of proactive therapy on sensitization in moderate to severe childhood atopic dermatitis: A randomized, investigator-blinded, controlled study. The Journal of dermatology. 2016.

22. Fukuie T, Nomura I, Horimukai K, Manki A, Masuko I, Futamura M, et al. Proactive treatment appears to decrease serum immunoglobulin-E levels in patients with severe atopic dermatitis. The British journal of dermatology. 2010;163(5):1127-9.

23. 山﨑 晃, 竹村 豊, 長井 恵, 井上 徳, 竹村 司. 〈原著〉食物アレルギー発症予防を目的とした乳児期早期の湿疹に対するプロアクティブ療法の効果：後方視的ケースコントロールスタディ. 近畿大学医学雑誌 = Medical Journal of Kindai University. 2016;41(1):9-16.

24. Callen J, Chamlin S, Eichenfield LF, Ellis C, Girardi M, Goldfarb M, et al. A systematic review of the safety of topical therapies for atopic dermatitis. The British journal of dermatology. 2007;156(2):203-21.

25. Eichenfield LF, Tom WL, Berger TG, Krol A, Paller AS, Schwarzenberger K, et al. Guidelines of care for the management of atopic dermatitis: section 2. Management and treatment of atopic dermatitis with topical therapies. Journal of the American Academy of Dermatology. 2014;71(1):116-32.

26. Ellison JA, Patel L, Ray DW, David TJ, Clayton PE. Hypothalamic-pituitary-adrenal function and glucocorticoid sensitivity in atopic dermatitis. Pediatrics. 2000;105(4 Pt 1):794-9.

27. Ebisawa M, Ito K, Fujisawa T. Japanese guidelines for food allergy 2017. Allergology International.66(2):248-64.

28. Warren CM, Otto AK, Walkner MM, Gupta RS. Quality of Life Among Food Allergic Patients and Their Caregivers. Current allergy and asthma reports. 2016;16(5):38.

29. Prescott S, Allen KJ. Food allergy: riding the second wave of the allergy epidemic. Pediatric allergy and immunology. 2011;22(2):155-60.

30. Ebisawa M, Nishima S, Ohnishi H, Kondo N. Pediatric allergy and immunology in Japan. Pediatric allergy and immunology : official publication of the European Society of Pediatric Allergy and Immunology. 2013;24(7):704-14.

31. Nwaru BI, Hickstein L, Panesar SS, Roberts G, Muraro A, Sheikh A. Prevalence of common food allergies in Europe: a systematic review and meta-analysis. Allergy. 2014;69(8):992-1007.

32. Hill DJ, Heine RG, Hosking CS, Brown J, Thiele L, Allen KJ, et al. IgE food sensitization in infants with eczema attending a dermatology department. The Journal of pediatrics. 2007;151(4):359-63.

33. Hon KL, Leung TF, Ching G, Chow CM, Luk V, Ko WS, et al. Patterns of food and aeroallergen sensitization in childhood eczema. Acta paediatrica (Oslo, Norway : 1992). 2008;97(12):1734-7.

34. Soderstrom L, Lilja G, Borres MP, Nilsson C. An explorative study of low levels of allergen-specific IgE and clinical allergy symptoms during early childhood. Allergy. 2011;66(8):1058-64.

35. Osborne NJ, Koplin JJ, Martin PE, Gurrin LC, Lowe AJ, Matheson MC, et al. Prevalence of challenge-proven IgE-mediated food allergy using population-based sampling and predetermined challenge criteria in infants. The Journal of allergy and clinical immunology. 2011;127(3):668-76 e1-2.

36. Kawamoto N, Fukao T, Kaneko H, Hirayama K, Sakurai S, Arai T, et al. Risk factors for infantile atopic dermatitis and recurrent wheezing. Journal of investigational allergology & clinical immunology. 2012;22(2):116-25.

37. Moghtaderi M, Farjadian S, Kashef S, Alyasin S, Afrasiabi M, Orooj M. Specific IgE to common food allergens in children with atopic dermatitis. Iranian journal of immunology : IJI. 2012;9(1):32-8.

38. Martin PE, Eckert JK, Koplin JJ, Lowe AJ, Gurrin LC, Dharmage SC, et al. Which infants with eczema are at risk of food allergy? Results from a population-based cohort. Clinical and experimental allergy : journal of the British Society for Allergy and Clinical Immunology. 2015;45(1):255-64.

39. Lack G, Fox D, Northstone K, Golding J. Factors associated with the development of peanut allergy in childhood. The New England journal of medicine. 2003;348(11):977-85.

40. van den Oord RA, Sheikh A. Filaggrin gene defects and risk of developing allergic sensitisation and allergic disorders: systematic review and meta-analysis. BMJ (Clinical research ed). 2009;339:b2433.

41. Flohr C, Perkin M, Logan K, Marrs T, Radulovic S, Campbell LE, et al. Atopic dermatitis and disease severity are the main risk factors for food sensitization in exclusively breastfed infants. The Journal of investigative dermatology. 2014;134(2):345-50.

42. Spergel JM, Boguniewicz M, Schneider L, Hanifin JM, Paller AS, Eichenfield LF. Food Allergy in Infants With Atopic Dermatitis: Limitations of Food-Specific IgE Measurements. Pediatrics. 2015;136(6):e1530-8.

43. Du Toit G, Katz Y, Sasieni P, Mesher D, Maleki SJ, Fisher HR, et al. Early consumption of peanuts in infancy is associated with a low prevalence of peanut allergy. The Journal of allergy and clinical immunology. 2008;122(5):984-91.

44. Du Toit G, Roberts G, Sayre PH, Bahnson HT, Radulovic S, Santos AF, et al. Randomized trial of peanut consumption in infants at risk for peanut allergy. The New England journal of medicine. 2015;372(9):803-13.

45. Perkin MR, Logan K, Tseng A, Raji B, Ayis S, Peacock J, et al. Randomized Trial of Introduction of Allergenic Foods in Breast-Fed Infants. The New England journal of medicine. 2016;374(18):1733-43.

46. Bellach J, Schwarz V, Ahrens B, Trendelenburg V, Aksunger O, Kalb B, et al. Randomized placebo-controlled trial of hen's egg consumption for primary prevention in infants. The Journal of allergy and clinical immunology. 2016.

47. Palmer DJ, Sullivan TR, Gold MS, Prescott SL, Makrides M. Randomized controlled trial of early regular egg intake to prevent egg allergy. The Journal of allergy and clinical immunology. 2016.

48. Natsume O, Kabashima S, Nakazato J, Yamamoto-Hanada K, Narita M, Kondo M, et al. Two-step egg introduction for prevention of egg allergy in high-risk infants with eczema (PETIT): a randomised, double-blind, placebo-controlled trial. The Lancet.

49. Lack G. Epidemiologic risks for food allergy. The Journal of allergy and clinical immunology. 2008;121(6):1331-6.

50. Urisu A, Ebisawa M, Ito K, Aihara Y, Ito S, Mayumi M, et al. Japanese Guideline for Food Allergy 2014. Allergology international : official journal of the Japanese Society of Allergology. 2014;63(3):399-419.

51. Wood RA. Food allergen immunotherapy: Current status and prospects for the future. The Journal of allergy and clinical immunology. 2016;137(4):973-82.

52. Caminiti L, Pajno GB, Crisafulli G, Chiera F, Collura M, Panasci G, et al. Oral Immunotherapy for Egg Allergy: A Double-Blind Placebo-Controlled Study, with Postdesensitization Follow-Up. The journal of allergy and clinical immunology In practice. 2015;3(4):532-9.

53. Baba M, Yamaguchi K. The allergy march”: Can it be prevented. Allergy Clin Immunol News. 1989;1:71-3.

54. 文部科学省. 「学校生活における健康管理に関する調査」中間報告 平成25年12月16日.

55. Williams HC, Burney PG, Hay RJ, Archer CB, Shipley MJ, Hunter JJ, et al. The U.K. Working Party's Diagnostic Criteria for Atopic Dermatitis. I. Derivation of a minimum set of discriminators for atopic dermatitis. The British journal of dermatology. 1994;131(3):383-96.

56. Williams HC, Burney PG, Strachan D, Hay RJ. The U.K. Working Party's Diagnostic Criteria for Atopic Dermatitis. II. Observer variation of clinical diagnosis and signs of atopic dermatitis. The British journal of dermatology. 1994;131(3):397-405.

57. Williams HC, Burney PG, Pembroke AC, Hay RJ. The U.K. Working Party's Diagnostic Criteria for Atopic Dermatitis. III. Independent hospital validation. The British journal of dermatology. 1994;131(3):406-16.

58. Lewis-Jones MS, Finlay AY, Dykes PJ. The Infants' Dermatitis Quality of Life Index. The British journal of dermatology. 2001;144(1):104-10.

59. Basra MK, Gada V, Ungaro S, Finlay AY, Salek SM. Infants' Dermatitis Quality of Life Index: a decade of experience of validation and clinical application. The British journal of dermatology. 2013;169(4):760-8.

60. Lawson V, Lewis-Jones MS, Finlay AY, Reid P, Owens RG. The family impact of childhood atopic dermatitis: the Dermatitis Family Impact Questionnaire. The British journal of dermatology. 1998;138(1):107-13.

61. Hanifin JM, Thurston M, Omoto M, Cherill R, Tofte SJ, Graeber M. The eczema area and severity index (EASI): assessment of reliability in atopic dermatitis. EASI Evaluator Group. Experimental dermatology. 2001;10(1):11-8.

62. Schmitt J, Spuls PI, Thomas KS, Simpson E, Furue M, Deckert S, et al. The Harmonising Outcome Measures for Eczema (HOME) statement to assess clinical signs of atopic eczema in trials. The Journal of allergy and clinical immunology. 2014;134(4):800-7.

63. Charman CR, Venn AJ, Williams HC. The patient-oriented eczema measure: Development and initial validation of a new tool for measuring atopic eczema severity from the patients’ perspective. Arch Dermatol. 2004;140(12):1513-9.

64. Chalmers JR, Simpson E, Apfelbacher CJ, Thomas KS, von Kobyletzki L, Schmitt J, et al. Report from the fourth international consensus meeting to harmonize core outcome measures for atopic eczema/dermatitis clinical trials (HOME initiative). The British journal of dermatology. 2016.

65. Sampson HA, Gerth van Wijk R, Bindslev-Jensen C, Sicherer S, Teuber SS, Burks AW, et al. Standardizing double-blind, placebo-controlled oral food challenges: American Academy of Allergy, Asthma & Immunology-European Academy of Allergy and Clinical Immunology PRACTALL consensus report. The Journal of allergy and clinical immunology. 2012;130(6):1260-74.

66. Simpson EL, Keck LE, Chalmers JR, Williams HC. How should an incident case of atopic dermatitis be defined? A systematic review of primary prevention studies. The Journal of allergy and clinical immunology. 2012;130(1):137-44.

67. Palmer DJ, Metcalfe J, Makrides M, Gold MS, Quinn P, West CE, et al. Early regular egg exposure in infants with eczema: A randomized controlled trial. The Journal of allergy and clinical immunology. 2013;132(2):387-92 e1.
